# Positive feedback of SuFu negating protein 1 on Hedgehog signaling promotes colorectal tumor growth

**DOI:** 10.1038/s41419-021-03487-0

**Published:** 2021-02-19

**Authors:** Zhengwei Yan, Minzhang Cheng, Guohui Hu, Yao Wang, Shaopeng Zeng, Aidi Huang, Linlin Xu, Yuan Liu, Chao Shi, Libin Deng, Quqin Lu, Hai Rao, Hua Lu, Ye-Guang Chen, Shiwen Luo

**Affiliations:** 1 Center for Experimental Medicine, The First Affiliated Hospital of Nanchang University, 330006 Nanchang, Jiangxi China; 2 The State Key Laboratory of Membrane Biology, Tsinghua-Peking Center for Life Sciences, School of Life Sciences, Tsinghua University, 100084 Beijing, China; 3 Basic Medical College, Nanchang University, 330006 Nanchang, Jiangxi China; 4 Department of Biostatistics and Epidemiology, School of Public Health, Nanchang University, 330006 Nanchang, Jiangxi China; 5 Department of Molecular Medicine, The University of Texas Health, San Antonio, TX 78229 USA; 6 Department of Biochemistry and Molecular Biology, Tulane University School of Medicine, New Orleans, LA 70112 USA; 7 Tulane Cancer Center, Tulane University School of Medicine, New Orleans, LA 70112 USA; 8 Present Address: Guangzhou Jinan Biomedicine Research and Development Center, College of Life Science and Technology, Jinan University, 510632 Guangzhou, Guangdong China

**Keywords:** Oncogenes, Growth factor signalling

## Abstract

Hedgehog (Hh) signaling plays a critical role in embryogenesis and tissue homeostasis, and its deregulation has been associated with tumor growth. The tumor suppressor SuFu inhibits Hh signaling by preventing the nuclear translocation of Gli and suppressing cell proliferation. Regulation of SuFu activity and stability is key to controlling Hh signaling. Here, we unveil **S**uFu **Ne**gating **P**rotein 1 (SNEP1) as a novel Hh target, that enhances the ubiquitination and proteasomal degradation of SuFu and thus promotes Hh signaling. We further show that the E3 ubiquitin ligase LNX1 plays a critical role in the SNEP1-mediated degradation of SuFu. Accordingly, SNEP1 promotes colorectal cancer (CRC) cell proliferation and tumor growth. High levels of SNEP1 are detected in CRC tissues and are well correlated with poor prognosis in CRC patients. Moreover, SNEP1 overexpression reduces sensitivity to anti-Hh inhibitor in CRC cells. Altogether, our findings demonstrate that SNEP1 acts as a novel feedback regulator of Hh signaling by destabilizing SuFu and promoting tumor growth and anti-Hh resistance.

## Introduction

Deregulation of Hedgehog (Hh) signaling, which is essential for cell proliferation and differentiation in embryonic development, often results in multiple developmental defects^1,2^. Hh signaling is initiated by Hh ligands, including Sonic hedgehog (Shh), Indian hedgehog (Ihh), or Desert hedgehog (Dhh), followed by activation of its downstream cascade that comprises Patched homolog (PTCH), Smoothened homolog (SMO), Suppressor of Fused homolog (SuFu), and the family of Gli transcriptional factors. Among the three Gli homologs, Gli2 and Gli3 can undergo partial proteolysis to generate a suppressive form in the absence of Hh ligands, while Gli1 lacks this suppressive domain^3^.

Because of its key role in cell fate determination, Hh signaling is tightly regulated by both positive and negative feedback mechanisms. Specifically, Gli1 upregulates the expression of its target genes in response to Hh ligands in a positive feedback fashion^4^. Additionally, Hh signaling can be self-controlled by activating the expression of PTCH, an upstream suppressor in the pathway^5^. While the Hh pathway normally regulates cell proliferation and differentiation, its dysregulation promotes tumor formation and progression^6,7^. A number of oncogenes, such as *Sox2*, *c-Myc*, *Bcl2*, *FoxM1*, etc.^8^, have been identified as Hh targets. Overexpression of these oncogenes due to deregulation of Hh signaling plays critical roles in the initiation and maintenance of multiple types of tumors, such as melanoma^9,10^, glioblastoma^11^, and colorectal cancer (CRC)^12–14^. Dysregulation of the Hedgehog signaling pathway plays a critical role in colorectal cancer (CRC) growth, but its molecular mechanism remains unclear.

SuFu, a PEST-domain protein, serves as a key negative regulator of Hh signaling by preventing the nuclear accumulation of Gli transcriptional factors. It plays an essential role in embryonic development, as knocking out the SuFu gene results in early embryonic lethality at gestation with neural tube defects^15^. Conditional SuFu knockout leads to abnormal cardiac looping^16,17^ and other developmental defects^18,19^. Additionally, SuFu can function as a tumor suppressor, as SuFu depletion accelerates tumorigenesis in TP53^−/−^ mice^20,21^. In humans, SuFu mutations are associated with Gorlin’s syndrome^22^, a hereditary disease with an increase incidence of tumors, such as basal cell carcinoma and medulloblastoma. Germline SuFu mutations have also been identified in these tumors^23–26^.

SuFu stability is tightly regulated. Hh activation induces SuFu degradation via the ubiquitination-proteasome pathway. Mono- and polyubiquitination of SuFu have been reported^27^. Two E3 ligase complexes, SCF^Fbxl17^ ^28^ and β-arrestin2-Itch^29^, have been reported to mediate the ubiquitination and turnover of SuFu. Its ubiquitination is further controlled by multiple post-translational modifications, such as phosphorylation by PKA and GSK3β^30^. However, it remains incompletely understood whether there are additional factors that regulate SuFu stability in response to Hh activation.

In this study, we identified *C18orf56* (GenBank: NM_001012716) as a novel Hh target gene. It is located at chromosome 18p11.32 and encodes a protein of 121 amino acid residues without any reported functions, although its transcription and translation have been verified via high-throughput screening^31^. In this study, we showed this protein as a SuFu suppressor and thus named it “**S**uFu **ne**gating **p**rotein **1**” (**SNEP1**). We showed that SNEP1 can promote SuFu degradation by interacting with an E3 ubiquitin ligase called ligand of numb-protein X1 (LNX1) and enhancing its activity toward SuFu in response to Hh activation. Additionally, SNEP1 is highly expressed in human CRCs, and this high expression is associated with poor prognosis. Thus, our study uncovers SNEP1 as a positive feedback regulator of the Hh signaling pathway, a crucial oncogenic player in colorectal cancer development and progression, and a potential drug target for the future development of anti-CRC therapy.

## Results

### SNEP1 is a downstream target of the Gli transcriptional factor

**Fig. 1:**
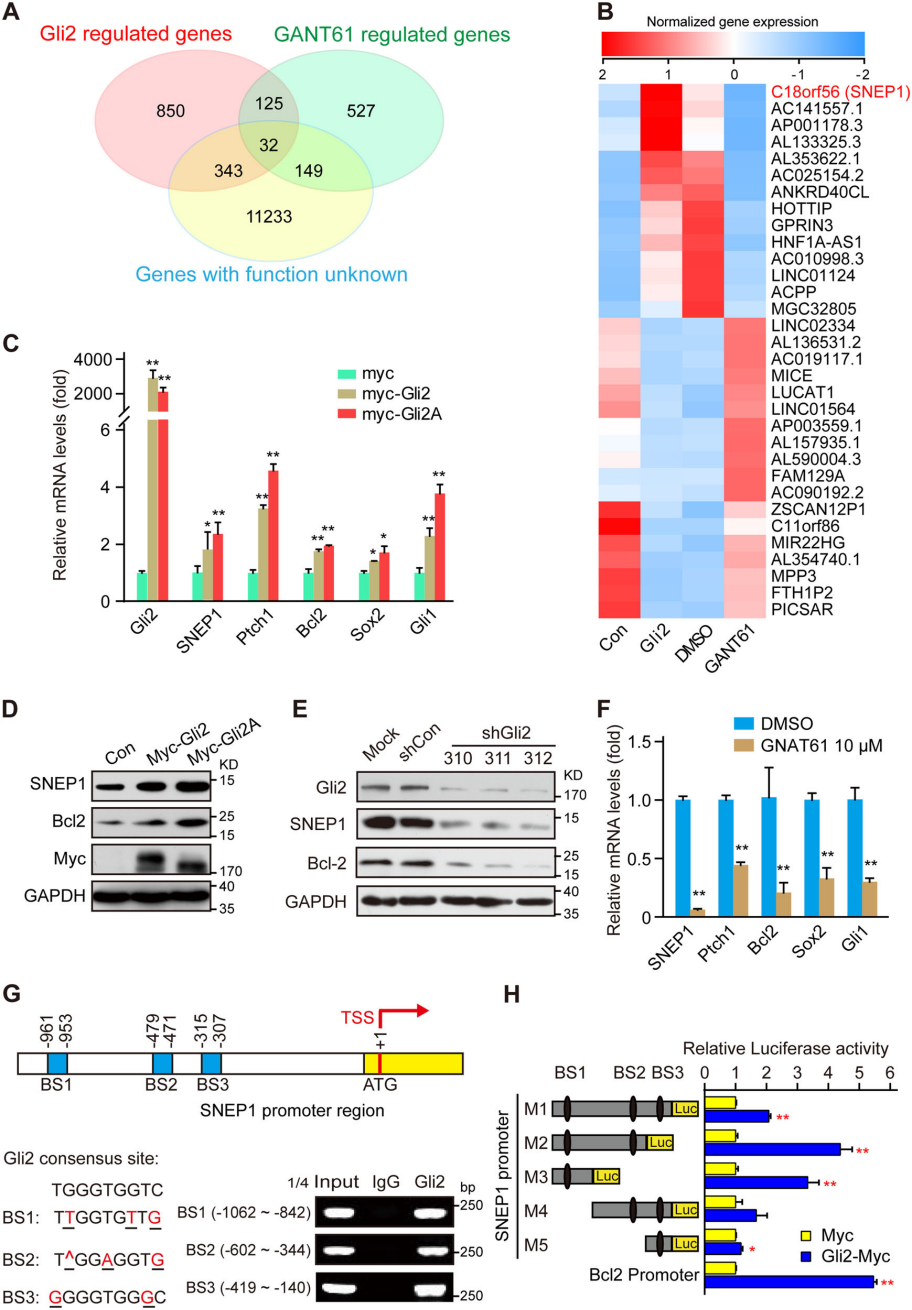
SNEP1 is a downstream target gene of the Gli transcriptional factor. **A**, **B** Screening for novel downstream target genes of Hh signaling. Venn diagram (**A**) and heatmap (**B**) of differentially expressed genes (DEGs) (fold change ≥2 or ≤ 0.05, adjusted *p* < 0.05) in HT-29 cells treated with GANT61 or expressing Gli2 and cluster analysis of these genes with Gene Ontology (GO) annotation. **C** Gli2 expression affects SNEP1 mRNA levels. HT-29 cells were transfected with Myc-vector, Myc-Gli2, or Myc-Gli2A for 48 h and harvested for qPCR analysis of SNEP1 mRNA. Bcl2, Ptch1, Sox2 and Gli1, well-known Hh signaling target genes, were used as positive controls. **D** Gli2 expression affects SNEP1 protein levels. HT-29 cells were transfected with Gli2 constructs for 48 h and harvested for IB with the indicated antibodies. **E** Gli2 knockdown reduces SNEP1 protein levels. HT-29 cells were transfected with shRNAi-Gli2 plasmids for 72 h and harvested for IB analysis with the indicated antibodies. **F** Pharmacological repression of Gli2 reduces SNEP1 mRNA levels. HT-29 cells were treated with GANT61 for 48 h and harvested for RNA extraction and qPCR. **G** Chromatin immunoprecipitation (ChIP) assays for Gli2 at the SNEP1 promoter. Upper: schematic representation of the SNEP1 promoter region showing the putative transcription factor-binding sites. Lower: HT-29 cells were harvested for ChIP assays as described in the Materials and Methods with IgG or anti-Gli2 antibodies for IP followed by qPCR with specific primers for each putative-binding element as indicated. **H** Luciferase assays for Gli2 transcriptional activity at the SNEP1 promoter. A series of SNEP1-luciferase constructs (left) were transfected into HEK-293T cells, and relative Gli2 transcript levels were measured 48 h after transfection (right). Each experiment was performed in triplicate. *p < 0.05, **p < 0.01.

To identify novel Gli-responsive genes, CRC HT-29 cells, which are widely used as Hh-responsive cells^32,33^, were treated with the small molecule Gli inhibitor GANT61 or subjected to ectopic expression of Gli2, and the gene expression profiles were determined by next-generation sequencing. Among 157 genes whose expression was dramatically regulated by both GANT61 and Gli2, 32 had no annotated function in the gene ontology (GO) database (Fig. 1A), and SNEP1 (C18orf56) attracted our interest (Fig. 1B). Interestingly, SNEP1 was also identified as a GANT61-regulated gene in previous high-throughput screening via cDNA microarray, which further confirmed our screening results^34^.

To confirm that SNEP1 is a Hh pathway target, we transfected different CRC cell lines with the Gli2 or Gli2A (constitutively activated mutation) overexpression construct, which increased SNEP1 messenger RNA (mRNA) (Fig. 1C) and protein (Fig. 1D) levels. In contrast, knocking down Gli2 reduced SNEP1 protein levels (Figs. 1E and S1A). Furthermore, blocking the transcriptional activity of Gli via GANT61 also decreased SNEP1 expression (Figs. 1F and S1B). These results suggest that SNEP1 expression is upregulated by the transcription factor Gli2.

Next, we determined whether SNEP1 is a direct target gene of Gli2. Bioinformatic analysis using MatInspector professional version 7.2^35^ from Genomatics (http://www.genomatix.de/) revealed three potential Gli-binding sites (BS1: −1062 ~ −842, BS2: −602 ~ −344, and BS3: −419 ~ −140) in the promoter region of SNEP1. In HT-29 cells, Gli2 was able to bind to all of these Gli-responsive DNA elements, as shown by chromatin immunoprecipitation (ChIP) analysis (Fig. 1G). Luciferase reporter assays driven by different fragments of the SNEP1 promoter region revealed that Gli2-binding site 1 (BS1) was the most important for Gli2 to regulate SNEP1 expression (Fig. 1H). Of note, the luciferase activity was lower when the reporter contained all of the BS motifs than when it contained BS1 only (Fig. 1H). This might be due to the possible competition for limited amounts of Gli2 molecules by multiple BS motifs, among which BS2 and BS3 were less effective. Taken together, these results demonstrate that Gli2 can directly induce SNEP1 expression at the transcriptional level.

### SNEP1 promotes CRC cell prolifer**a**tion and tumor growth

**Fig. 2:**
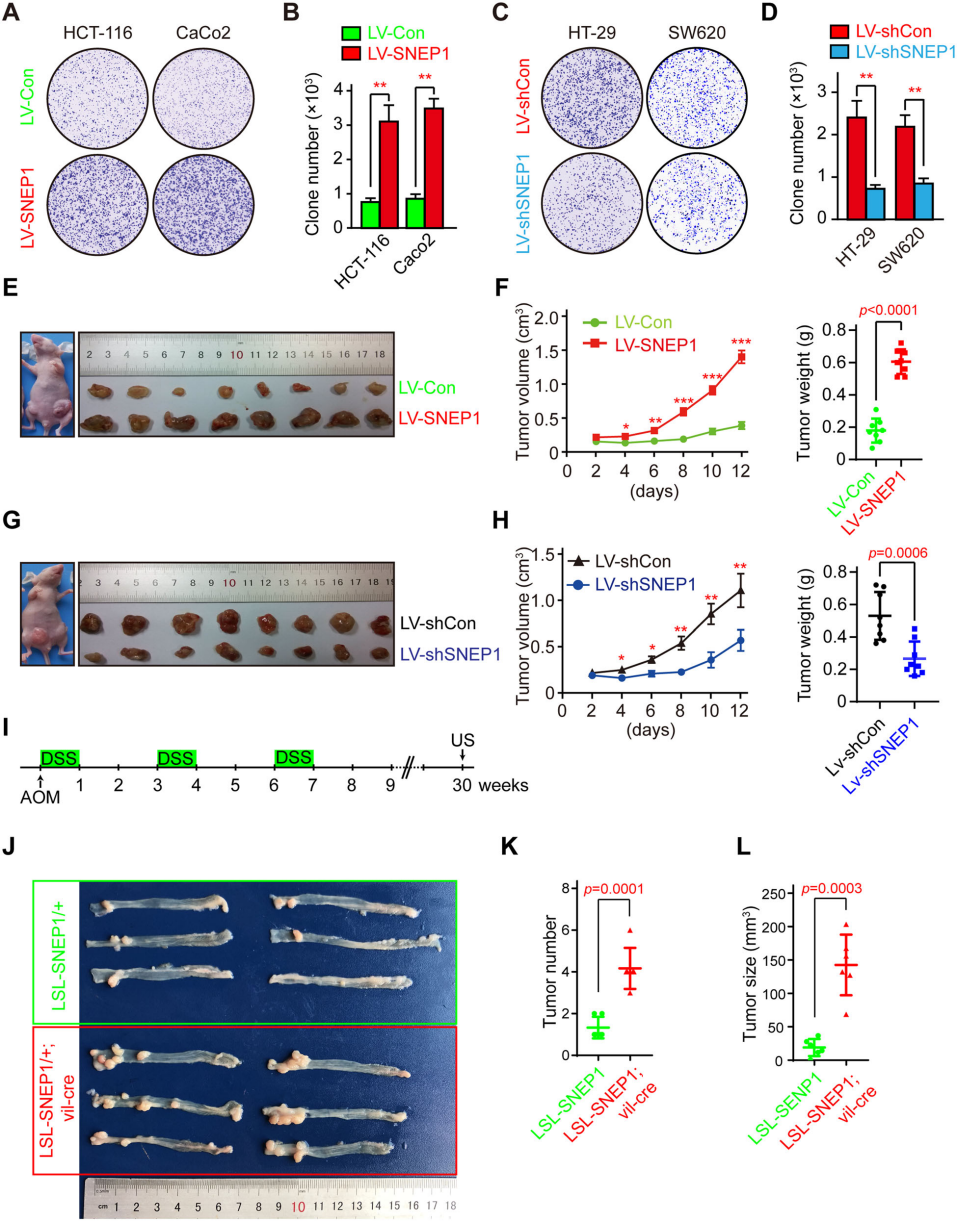
SNEP1 promotes CRC cell proliferation and tumor growth. **A** SNEP1 promotes the colony formation of CRC cells. HCT-116 or Caco2 cells stably expressing LV-SNEP1 were seeded in a 6-well plate for 14 days. The cells were then stained with 0.5% crystal violet (w/v). **B** Quantitative analysis of HCT-116 and Caco2 cells stably overexpressing SNEP1. The bar graph displays the means ± SD, *n* = 3, **p < 0.01. **C** SNEP1 knockdown inhibits colony formation of CRC cells. HT-29 or SW620 cells stably expressing LV-shSNEP1 were seeded in a 6-well plate for 14 days. The cells were then stained with 0.5% crystal violet (w/v). **D** Quantitative analysis of HT-29 and SW620 cell lines with stable SNEP1 knockdown was performed using ImageJ software. The bar graph displays the mean ± SD, *n* = 3, **p < 0.01. **E**, **F** SNEP1 overexpression promotes tumor growth ex vivo. HCT-116 stable cell lines (2 × 10^7^ cells) that overexpressed SNEP1 were subcutaneously injected into eight nude mice on each side of the inguinal region. Xenografts were harvested after 2 weeks. Tumor sizes on either side were monitored every other day and tumor weights are shown in **F**. Data are presented as mean ± SD (*n* = 8). **G**, **H** SNEP1 knockdown leads to suppression of tumor growth ex vivo. HT-29 stable cell lines (2 × 10^7^ cells) with LV-shSNEP1 were subcutaneously injected into eight nude mice in each side of the inguinal region. Xenografts were harvested after 2 weeks. Tumor sizes on either side were monitored every alternate day and the tumor weights were shown in **H**. Data are presented as mean ± SD (*n* = 8). **I** Schematic showing mouse treatment for the inflammation-dependent CRC model. **J**–**L** SNEP1 overexpression promotes tumor growth in an inflammation-dependent CRC model. Tumor sizes and weights are shown in **K** and **L**) Data are presented as the mean ± SD.

To determine the role of SNEP1 in CRC cell proliferation and growth, we detected the expression level of SNEP1 in various CRC cell lines (Fig. S2A) and used lentivirus to generate CRC cell lines with SNEP1 overexpression (HCT-116 and CaCo2 cells, which have a relatively low endogenous expression of SNEP1) or downregulation (HT-29 and SW620 cells, which have relatively high endogenous levels of SNEP1). Cell proliferation was evaluated by the colony formation assay, ectopic SNEP1 expression increased the number of HCT-116 and Caco2 cell colonies (Fig. 2A, B), while depletion of SNEP1 in HT-29 and SW620 cells reduced their colony formation ability (Fig. 2C, D). These results indicate that SNEP1 can promote CRC cell proliferation in vitro.

To determine the biological function of SNEP1 ex vivo, we established CRC xenograft mouse models using HCT-116 cells with inducible SNEP1 expression or HT-29 cells with inducible knockdown. Cells were injected subcutaneously into either flank of nude mice, and the tumor size was measured every two days. As shown in Fig. 2E, F, G, H, ectopic SNEP1 expression drastically increased tumor volume and weight, while SNEP1 knockdown decreased these values compared to those in the control groups. Next, we isolated xenografts and analyzed protein expression and cell proliferation by IB and immunohistochemistry (IHC). Ki67 staining showed an increased proliferative state in the presence of ectopic SNEP1 (Fig. S2B), while SNEP1 knockdown led to a reduce in Ki67 levels (Fig. S2C).

To further validate this, an inflammation-dependent colorectal cancer model^36^ was used to further investigate the biological function of SNEP1 in vivo. Azoxymethane (AOM) injection and dextran sulfate sodium (DSS) administration were used to induce colitis and adenoma in both LSL-SNEP1/+ and LSL-SNEP1/+ ;villin-cre/+ mice (Fig. 2I). Ectopic SNEP1 expression in the intestinal epithelium driven by villin-Cre facilitates colorectal adenoma growth in mice (Fig. 2J, K), and H&E staining further revealed that SNEP1 expression might lead to increased malignancy (Fig. S2F). Consistent with those in the ex vivo model, the Ki67 protein level was increased in SNEP1-expressing mice (Fig. S2F). Altogether, these results indicate that SNEP1 may promote CRC proliferation and tumor growth.

### SNEP1 activates the Hh signaling pathway by promoting the degradation of SuFu

**Fig. 3:**
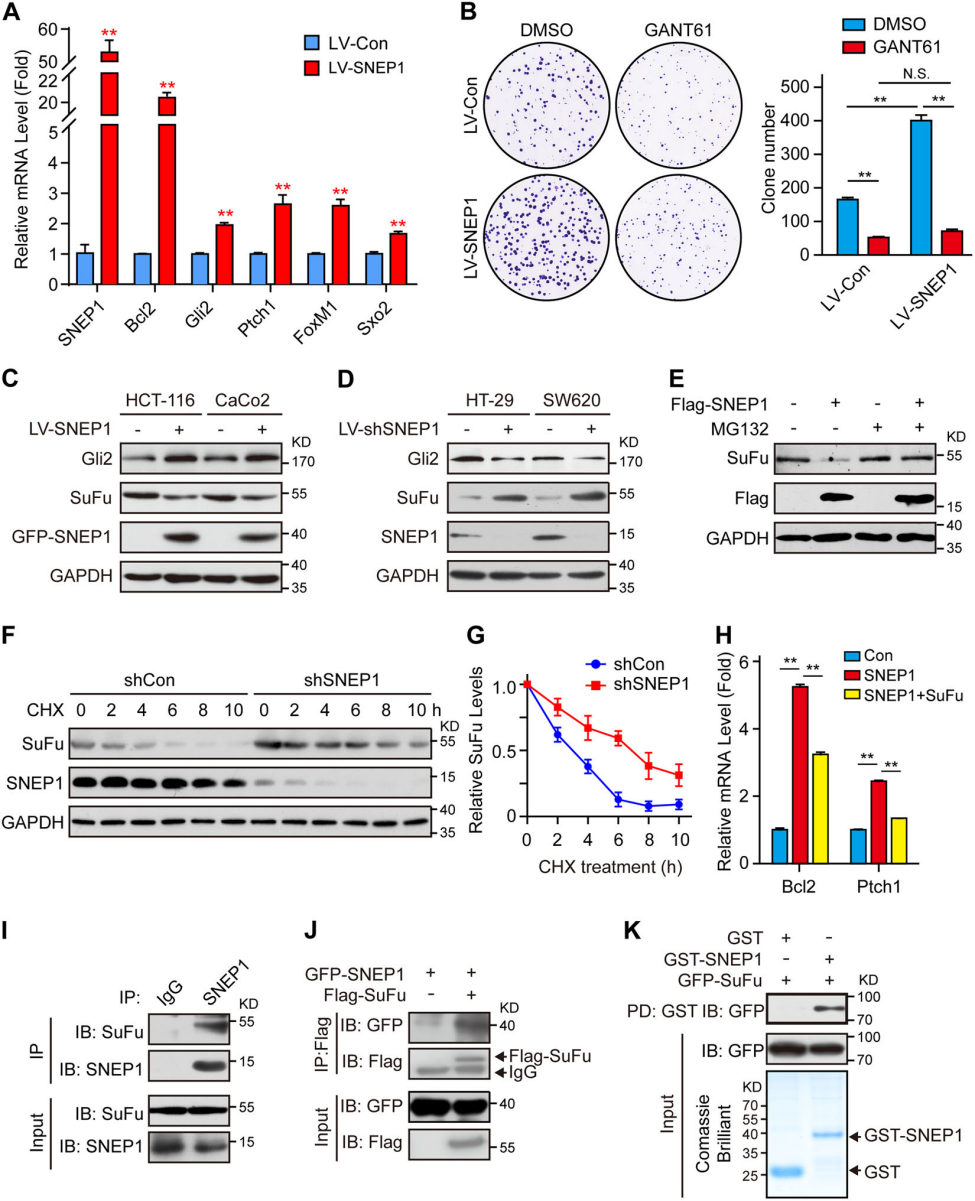
SNEP1-induced activation of Hh signaling and proliferation of CRC cell is mediated by SuFu degradation. **A** SNEP1 activates Hh signaling. HCT-116 cells with ectopic SNEP1 expression were harvested for qPCR. Data are presented as mean ± SD (*n* = 3). **p < 0.01. **B** SNEP1 facilitates CRC cell proliferation dependent on activation of the Hh Pathway. Caco2 cells were infected with LV-SNEP1 or control lentivirus, and treated with 2.5 μM GANT61. The cells were then stained with 0.5% crystal violet (w/v). The bar graph displays the mean ± SD, *n* = 3, **p < 0.01, N.S., not significance. **C** Exogenous expression of SNEP1 reduces SuFu protein levels. Protein levels of Gli2 and SuFu were detected via IB analysis of lysates isolated from lentivirus-transduced cell lines stably overexpressing SNEP1. **D** SNEP1 knockdown enhances SuFu protein levels. Protein levels of Gli2 and SuFu were measured by IB analysis of lysates isolated from lentivirus-transduced cells with stable SNEP1 knockdown. **E** MG132 rescues the reduction of SuFu protein levels by SNEP1. HEK-293T cells were transfected with a gradient Flag-SNEP1 construct for 48 h and were pretreated with the proteasome inhibitor MG132 overnight before harvesting. Lysates were examined via IB with the indicated antibodies. **F** SuFu degradation was attenuated by SNEP1 depletion. Cycloheximide (CHX) (100 μg/ml) was incubated for the indicated period with HEK-293T cells transfected with shRNA-SNEP1. Cell lysates were harvested for IB with anti-SuFu antibody. **G** Quantitative analysis of SuFu protein levels shown in **F** using ImageJ software. **H** Overexpression of SuFu decreased SNEP1-induced activation of Hh signaling. HCT-116 cells were infected with LV-SNEP1 or control lentivirus, and transfected with vector or SuFu plasmids for 48 h. qPCR were performed. Data are presented as mean ± SD (*n* = 3). **p < 0.01. **I** Endogenous complexes of SNEP1 with SuFu. Cell lysates of HT-29 cells were subjected to IP analysis with the indicated antibodies and protein-A/G beads overnight. Bound proteins were analyzed via IB. **J** Ectopically expressed SNEP1 interacts with SuFu. HEK-293T cells transfected with GFP-SNEP1 and Flag-SuFu plasmids were subjected to a Co-IP assay. **K** GST pull-down assay of SNEP1 and SuFu. The prokaryotically expressed GST-SNEP1 was incubated with cell lysates of GFP-SuFu-transfected HEK-293T cells. The protein complex pulled down by GST was detected via IB.

To study the mechanism of SNEP1 promoting the proliferation and growth of CRC, we performed a yeast two-hybrid screen using SNEP1 as bait. Interestingly, we identified SuFu, which is a negative regulator of the Hh signaling pathway, as one of the SNEP1-interacting proteins. We wondered whether SNEP1 as a downstream target gene may regulate Hh pathway activity. To evaluate this conjecture, the expression of downstream target genes of Hh signaling was checked, and the results showed that ectopic expression of SNEP1 increased both the mRNA (Fig. 3A) and protein levels (Fig. S3A) of classic Hh target genes, including Bcl2, Ptch1, Sox2, etc. Conversely, SNEP1 silence decreased the mRNA levels of classic Hh target genes (Fig. S3B). These results indicate that SNEP1 can activate the Hh signaling pathway. It is known that Hh signaling promotes cell proliferation and tumor growth in several types of cancers, including CRC (12). We therefore examined whether SNEP1 promotes CRC cell proliferation by activation of Hh signaling. We found that GANT61, a Gli inhibitor, can abrogate SNEP1-induced CRC cell proliferation (Figs. 3B and S3C), suggesting that SNEP1 promotes CRC cell proliferation via activation of Hh signaling.

Then, we evaluated whether SNEP1 activates the Hh pathway through regulating SuFu. We examined the level of SuFu protein in the constructed stable cell lines. Ectopic SNEP1 expression led to a decrease in SuFu protein levels but an increase in Gli2 protein levels in both HCT-116 and CaCo2 cells (Fig. 3C). Conversely, SNEP1 knockdown led to an increase in SuFu protein levels and a decrease in Gli2 protein levels in HT-29 and SW620 cells (Fig. 3D). Moreover, we detected the same result in vivo, as determined by immunohistochemistry and western blotting (Fig. S2B–G). These results suggest that SNEP1 can induce SuFu downregulation. When assessing their RNA levels, we found that interestingly, the RNA level of SuFu was not affected by ectopic SNEP1 in HCT-116 cells, while the Bcl2 mRNA level was induced by ectopic SNEP1 (Fig. S3D). These results suggest that SNEP1 may affect SuFu protein levels via post-translational mechanisms. Indeed, the proteasome inhibitor MG132 blocked the SNEP1-induced reduction in SuFu (Fig. 3E), suggesting that SNEP1 reduced SuFu levels through proteasome-mediated degradation. Consistently, blocking protein synthesis with cycloheximide (CHX) revealed that depleting SNEP1 stabilizes SuFu (Fig. 3F, G). Moreover, overexpression of SuFu decreased SNEP1-induced activation of Hh signaling (Fig. 3H). These results indicate that SNEP1 activates the Hh pathway through promoting the degradation of SuFu.

To determine whether SNEP1 regulates SuFu protein stability by directly binding to this protein, we performed coimmunoprecipitation (Co-IP) assays. As a result, both endogenous and exogenous SNEP1 and SuFu bound to each other in reciprocal Co-IP analyses in HT-29 or HEK-293T cells (Fig. 3I, J). This interaction was directly confirmed by an in vitro glutathione S-transferase (GST) pull-down assay with *E. coli*-expressed and purified GST-tagged SNEP1 (GST-SNEP1) (Fig. 3K). Domain mapping experiments revealed that the aa110–174 fragment of SuFu was essential for the interaction between SNEP1 and SuFu (Fig. S3E, F). Additionally, SNEP1 was pulled down by this aa110–174 fragment of SuFu (Fig. S3G), the truncation of aa110–174 in SuFu (Δ110–174) abolished the SNEP1-SuFu interaction (Fig. S3H). Further, the interacting fragment of SuFu (aa110–174) protected SuFu from degradation (Fig. S3I, J). Together with the data above, these results indicate that SNEP1 can regulate SuFu protein levels via direct interaction.

### SNEP1 enhances the int**e**raction between SuFu and LNX1

**Fig. 4:**
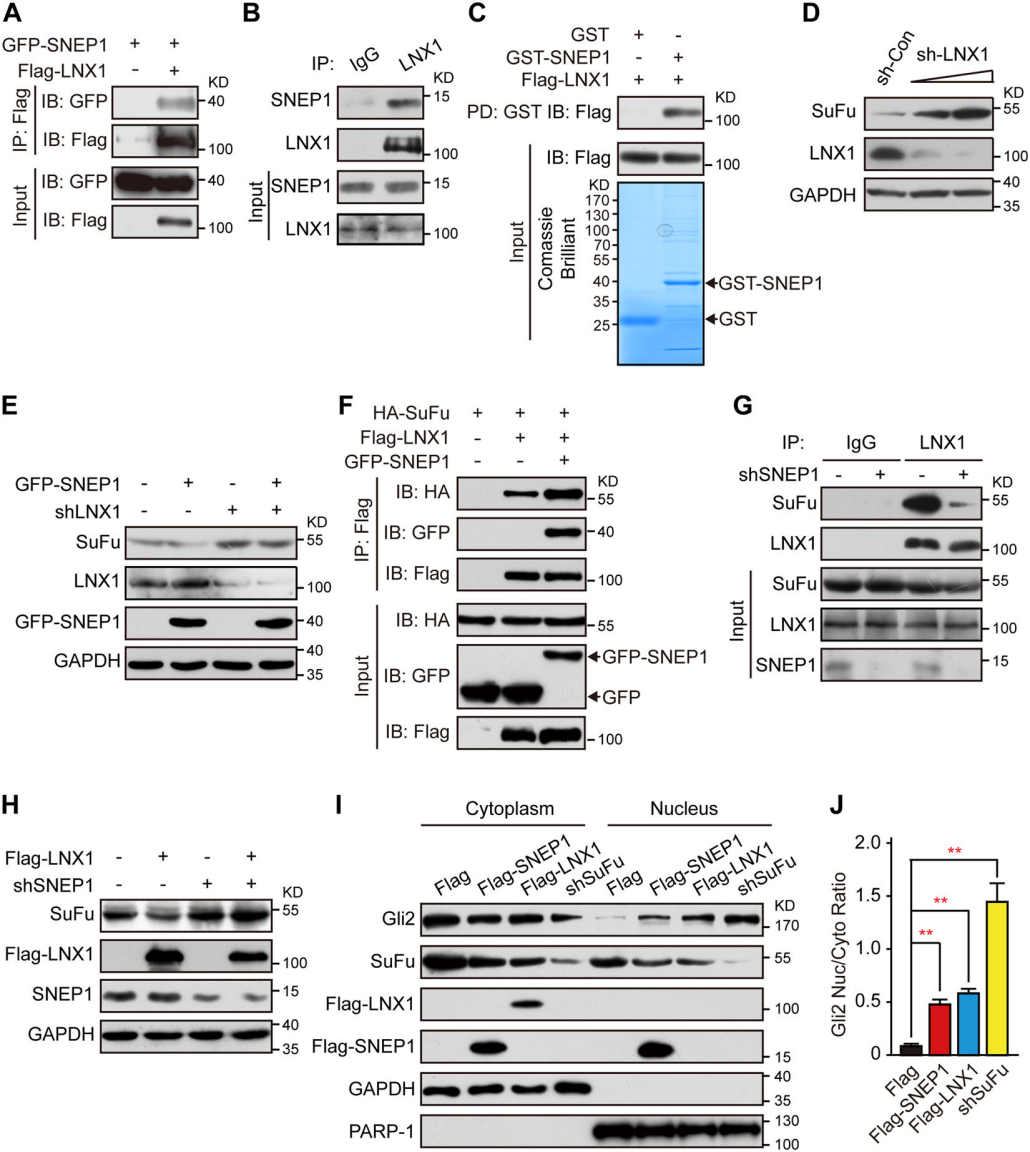
SNEP1 enhances LNX1-SuFu interactions and facilitates LNX1-mediated SuFu proteolysis. **A** An IP assay of SNEP1 with LNX1. After transfection with the indicated plasmids, HEK-293T cells were harvested for a Co-IP assay with an antibody recognizing Flag-tag, and binding proteins were detected via IB. **B** An IP assay of SNEP1 with LNX1. HT-29 cell lysates were subjected to a Co-IP assay with the indicated antibodies and protein-A/G beads overnight. Bound proteins were detected via IB. **C** GST pull-down assay of SNEP1 with LNX1. The prokaryotically expressed GST-SNEP1 was incubated with HEK-293T cell lysates overexpressing Flag-LNX1. The protein complexes pulled down with GST were detected via IB. **D** LNX1 knockdown increases SuFu protein levels. HEK-293T cells were transfected with different doses of sh-LNX1 construct for 48 h and harvested for IB analysis. **E** SNEP1-mediated SuFu degradation depends on LNX1. IB analysis of HEK-293T cell lysates that expressed GFP-SNEP1 with LNX1 knockdown or not. **F** Ectopic SNEP1 increases SuFu-LNX1 interactions. HEK-293T cells were cotransfected with HA-SuFu and Flag-LNX1 with or without GFP-SNEP1 plasmids and treated with MG132 overnight before harvesting. Cell lysates were subjected to a co-IP-IB assay. **G** SNEP1 is required for the interaction between SuFu and LNX1. HT-29 cells stably expressing LV-shSNEP1 were treated with MG132 overnight before harvest. Cell lysates were subjected to a co-IP-IB assay. **H** SNEP1 knockdown reverses the repression of the action of LNX1 on SuFu. IB analysis of HEK-293T cell lysates expressing Flag-LNX1 with or without shRNA-SNEP1. **I** SuFu knockdown promotes translocation of Gli2 into the nucleus. HCT-116 cells were transfected with Flag-SNEP1, Flag-LNX1 or shRNA-SuFu for 48 h and subjected to IB for detection of cytoplasmic and nuclear fractions of Gli2. GAPDH and PARP-1 were used as cytoplasmic and nuclear loading controls, respectively. **J** Quantification of **I** using ImageJ software. Data are shown as the mean ± SD, *n* = 3, **p < 0.01, ***p < 0.001.

To further understand how SNEP1 regulates SuFu stability, we conducted bioinformatics analysis of the SNEP1 amino acid sequence but could not find any conserved E3-like domain in this protein (data not shown). However, an E3 ligase LNX1 was found in the SNEP1-interacting proteins. LNX1 is a RING-finger E3 ubiquitin-protein ligase^37^ but has never been reported to regulate Hh signaling. We confirmed the interaction between SNEP1 and LNX1 by Co-IP assay and GST pull-down assay (Fig. 4A–C). Among the four PDZ domains of LNX1, the first PDZ domain contributed to the interaction between LNX1 and SNEP1 (Fig. S4A–D).

Next, we wanted to test whether LNX1 is required for the regulation of SuFu stability. As shown in Fig. S4E–G and Fig. 4D, ectopic LNX1 expression reduced SuFu protein levels in HEK-293T cells, while shRNA-mediated knockdown of LNX1 increased these levels. Moreover, blocking protein synthesis with cycloheximide (CHX) revealed that depleting LNX1 stabilized SuFu (Fig. S4H, I). We then evaluated the relationship between LNX1 and SNEP1. Interestingly, overexpression of LNX1 enhanced SNEP1-mediated degradation of SuFu (Fig. S4J), and knockdown of endogenous LNX1 impaired SNEP1-mediated degradation of SuFu in HEK-293T cells (Fig. 4E). Moreover, the SuFu mutation (Δ110–174), which did not interact with SNEP1, attenuated LNX1-mediated degradation (Fig. S4K). Then, we assessed whether SNEP1 could affect the complex formation between LNX1 and SuFu. As shown in Fig. 4F, ectopic LNX1 and SuFu bound to each other, and this complex was elevated by SNEP1. Conversely, knockdown of SNEP1 impaired the LNX1-SuFu interaction (Fig. 4G) and concurrently rescued the LNX1-mediated SuFu reduction (Fig. 4H). Furthermore, we found that SNEP1 silence inhibited the LNX1-SuFu interaction, which enhanced by overexpression of Gli2 (Fig. S4L). These results indicate that SNEP1 acts as an adaptor protein to enhance the SuFu-LNX1 interaction and thus facilitates LNX1-mediated SuFu degradation.

SuFu attenuates Hh signaling by downregulating full-length Gli transcription factors and preventing their nuclear import^38,39^. Therefore, we assessed the nuclear distribution of Gli2 when LNX1 or SNEP1 was overexpressed in HCT-116 cells. As shown in Fig. 4I, J, overexpression of either SNEP1 or LNX1 resulted in a decrease in SuFu levels and consequently in an increase in nuclear Gli2. These results indicate that SNEP1 promotes the nuclear accumulation of Gli2 by mediating SuFu degradation.

### SNEP1 promotes LNX1-m**e**diated SuFu ubiquitination

**Fig. 5:**
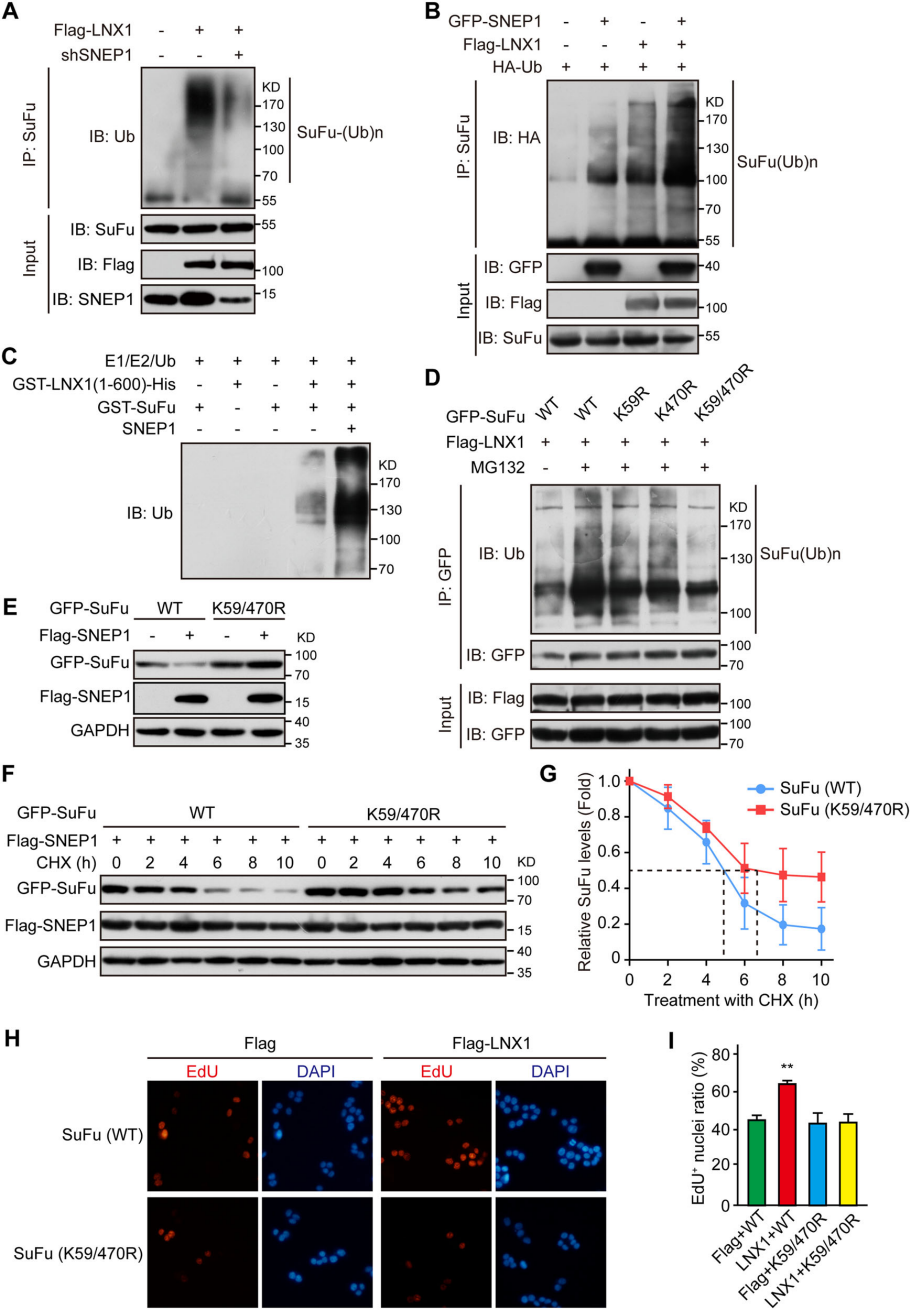
SNEP1 facilitates LNX1-mediated ubiquitination of SuFu. **A** SNEP1 knockdown impairs LNX1-mediated ubiquitination of SuFu. HEK-293T cells were transfected with Flag-LNX1, shRNA control, or shSNEP1 for 48 h and treated with MG132 overnight before harvest. Cell lysates were subjected to IP with anti-SuFu antibody and protein A/G beads overnight. Polyubiquitin chains bound to SuFu were assessed via IB. **B** SNEP1 increases LNX1-mediated ubiquitination of SuFu. HEK-293T cells were transfected with Flag-LNX1, GFP-vector, or GFP-SNEP1 for 48 h and treated with MG132 overnight before harvest. Cell lysates were subjected to IP with anti-SuFu antibody and protein A/G beads overnight. Polyubiquitin chains bound to SuFu were assessed via IB. **C** SNEP1 enhances LNX1-mediated SuFu ubiquitination in vitro. GST-SuFu, GST-LNX1 (1-600), and SNEP1 were expressed in and purified from *E. coli*. Purified proteins were mixed with E1, E2, and ubiquitin purchased from Enzo and incubated at 37 °C for 4 h. Polyubiquitin chains were assessed via IB. **D** The SNEP1-LNX1 complex targets K59 and K470 for SuFu ubiquitination. HEK-293T cells were transfected with GFP-SuFu, GFP-SuFu (K59R), GFP-SuFu (K470R), or GFP-SuFu (K59/470R) and Flag-LNX1 and treated with MG132 overnight before harvest. Cell lysates were subjected to IP with an anti-SuFu antibody and protein A/G beads overnight. Polyubiquitin chains bound to SuFu were assessed via IB. **E** SuFu (K59/470R) is resistant to degradation mediated by SNEP1. HEK-293T cells were transfected with GFP-SuFu or GFP-SuFu (K59/470R) and Flag-vector or SNEP1 for 48 h before harvest. Cell lysates were assessed via IB. **F** SuFu (K59/470R) displays a prolonged half-life in cells. CHX (100 μg/ml) was incubated for different time points with GFP-SuFu or GFP-SuFu (K59/470R) and Flag-SNEP1-transfected HEK-293T cells. Cell lysates were harvested for IB with the indicated antibodies. **G** Quantitative analysis of SuFu protein levels shown in **F** using ImageJ software. **H** SuFu (K59/470R) is inactivated in response to LNX1-promoted cell proliferation. HT-29 cells transfected with GFP-SuFu or GFP-SuFu (K59/470R) were treated with EdU for 4 h before fixation with paraformaldehyde. Cells were then stained with rhodamine-tagged anti-EdU antibody and DAPI. **I** Quantitative analysis of the ratio of EdU^+^ cells shown in **H**, **p < 0.01.

We tested whether LNX1 is required for SuFu ubiquitination. Indeed, LNX1 mediated the ubiquitination of SuFu (Fig. 5A), and LNX1 knockdown decreased the level of polyubiquitination of SuFu (Fig. S5A). Consistent with its role in facilitating the LNX1-SuFu interaction in HEK-293T cells, SNEP1 knockdown also reduced LNX1-mediated SuFu ubiquitination (Fig. 5A), while SNEP1 overexpression enhanced it in the presence of LNX1 (Fig. 5B). To further verify whether SNEP1 and LNX1 work together to mediate SuFu ubiquitination, LNX1-mediated SuFu ubiquitination was performed in vitro with purified SNEP1, SuFu, and the fragment of LNX1 containing E3 ligase activity (Fig. S5B). As shown in Fig. 5C, LNX1 ubiquitinated SuFu, which was greatly enhanced by SNEP1.

In the human SuFu protein, four of 15 lysines, K59, K398, K467, and K470, are evolutionally conserved from *Drosophila* to vertebrates (Fig. S5C). To assess whether these residues are ubiquitination sites, we generated point mutations with individual substitutions of these residues to arginine (K59R, K398R, K467R, or K470R). We found that SuFu-K59R and SuFu-K470R are resistant to LNX1-mediated degradation (Fig. S5D), suggesting that these two sites might be ubiquitination sites. Consistent with this, although the ubiquitination of each of the SuFu mutants by LNX1 was partially reduced, the ubiquitination of SuFu-K59R/470R by LNX1 was almost completely blocked (Fig. 5D). Additionally, the SNEP1- or LNX1-mediated degradation of this double mutant was completely blocked (Figs. 5E and S5E). Consistently, the half-life of SuFu-K59R/470 R was markedly prolonged even in the presence of SNEP1 or LNX1 expression (Figs. 5F, G and S5F). In line with these biochemical results, EdU labeling revealed that LNX1 failed to promote the proliferation of SuFu-K59R/K470R-expressing HT-29 cells (Fig. 5H, I). Taken together, these results demonstrate that LNX1 mediates ubiquitin conjugation at K59 and K470 of SuFu, which is essential for ubiquitin-dependent proteolysis of SuFu and for LNX1-promoted cell proliferation.

### SNEP1 is highly expressed in human CRC and predicts a poor clinical outcome

**Fig. 6:**
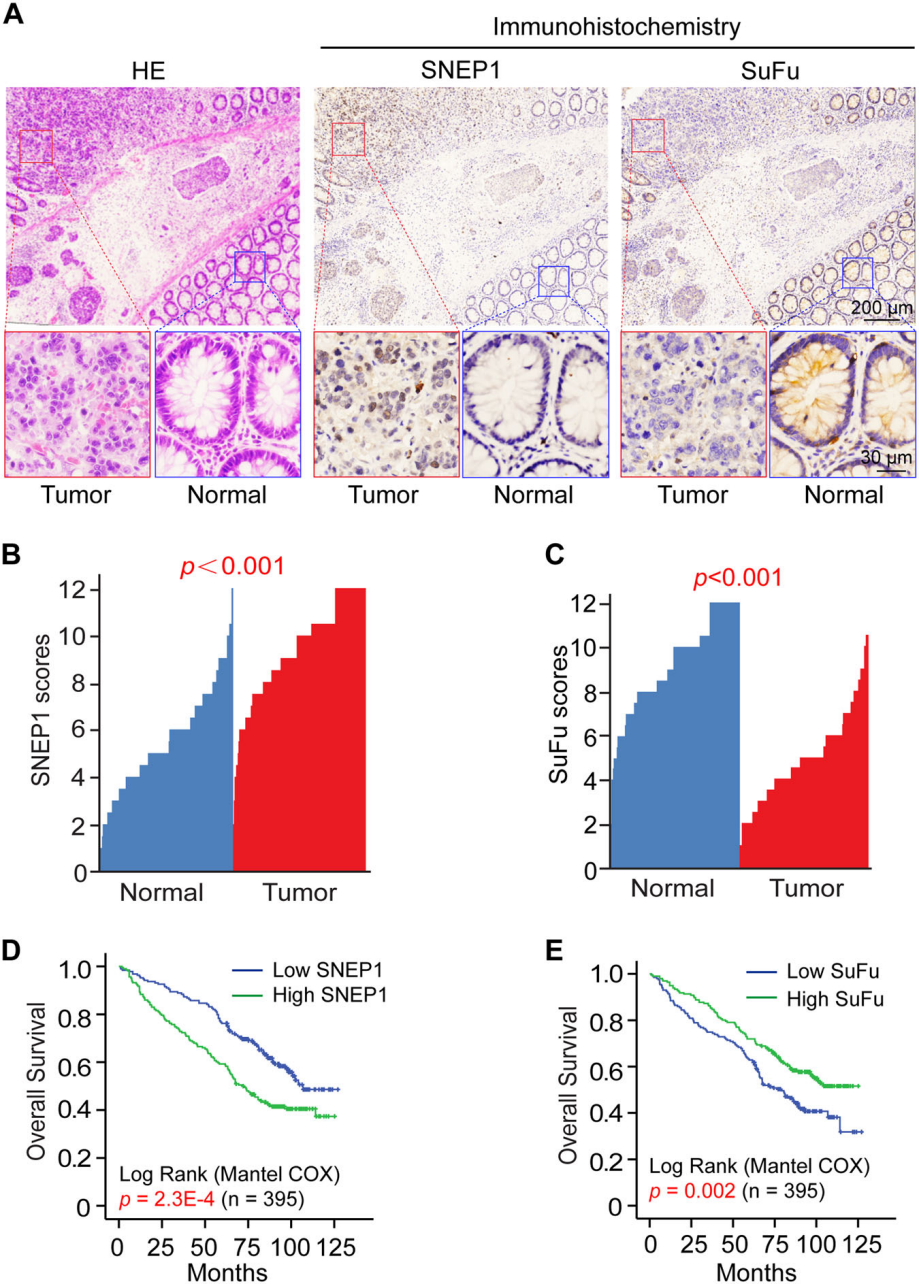
SNEP1 expression is elevated, but SuFu is decreased in primary CRC tissues. **A** Representative images of IHC staining of both human CRC tissues and adjacent tissues in the same section stained for SNEP1 and SuFu. **B**, **C** SNEP1 (**B**) and SuFu (**C**) expression was plotted per the IHC scores in each carcinoma and adjacent tissue. **D**–**E** Kaplan–Meier estimates of the overall survival of CRC patients between the negative/low and medium/high expression groups for SNEP1 (**D**) and SuFu (**E**).

To translate the aforementioned findings into clinical significance, we examined SNEP1 expression in primary CRC tumors. In total, 395 CRC samples with matched adjacent normal tissues were collected and examined via IHC analysis with specific anti-SNEP1 and anti-SuFu antibodies. Compared to the matched adjacent normal tissues, SNEP1 expression was greater in cancer tissues, accompanied by relatively lower expression of SuFu (Fig. 6A–C). In addition, higher pathological grades were associated with increased SNEP1 expression and lower SuFu expression (Fig. S6A–C). Furthermore, correlation analysis of expression revealed that SuFu expression was inversely correlated with SNEP1 in CRC, not correlated with LNX1 (Fig. S6D–E). Together, these results suggest that the SNEP1 level is inversely correlated with the SuFu level and that SNEP1 may function as an oncogenic protein in human CRC progression.

Furthermore, we analyzed the correlation between the expression of SNEP1 or SuFu and the pathological features of CRC tissues. We categorized tissue samples into two groups according to the expression of SNEP1 and SuFu based on their mean IHC staining scores using the German semiquantitative scoring system with consideration of both staining intensity and area. As shown in Table 1, high SNEP1 levels was positively correlated with poor histological grade, larger primary tumor size, general type and advanced Dukes stage in CRC, while low SuFu levels were associated with tumor location, positive vascular invasion, poor histological grade and late Dukes stage.

**Table 1:** Association of SNEP1 and SuFu expression levels with different clinicopathologic characteristics in CRC.

Clinicopathologic	SNEP1 expression		*p*-value	SuFu expression		*p*-value
	Low	High		Low	High	
	Count (*n*%)	Count (*n*%)		Count (*n*%)	Count (*n*%)	
Sex						
Male	106 (56.1)	112 (54.4)	0.732	110 (55.3)	18 (55.1)	0.972
Female	83 (43.9)	94 (45.6)		89 (44.7)	88 (44.9)	
Age, years						
<60	86 (45.5)	103 (50.0)	0.371	86 (43.2)	103 (52.6)	0.063
≥60	103 (54.5)	103 (50.0)		113 (56.8)	93 (47.4)	
CEA level, ng/ml						
≤5	90 (47.6)	103 (50.0)	0.167	103 (51.8)	90 (45.9)	0.233
>5	76 (40.2)	67 (32.5)		72 (36.2)	71 (36.2)	
Unknown	23 (12.2)	36 (17.5)		24 (12.1)	35 (17.9)	
CA-199, U/ml						
≤27	115 (60.8)	120 (58.3)	0.734	138 (69.3)	126 (64.3)	0.147
>27	37 (19.6)	39 (18.9)		22 (11.1)	16 (8.2)	
Unknown	37 (19.6)	47 (22.8)		39 (19.6)	54 (27.5)	
CA-125, U/ml						
≤34	137 (72.5)	127 (61.7)	0.053	131 (69.3)	133 (64.5)	0.143
>34	13 (6.9)	25 (12.1)		22 (11.6)	16 (7.8)	
Unknown	39 (20.6)	54 (26.2)		36 (19.1)	57 (27.7)	
General type						
Ulcer type	3 (1.6)	3 (1.5)	0.005**	3 (1.5)	3 (1.5)	0.519
Bulge type	118 (62.4)	158 (76.7)		144 (72.4)	132 (67.3)	
Other type	68 (36.0)	45 (21.8)		52 (26.1)	61 (31.2)	
History of intestinal polyps						
Negative	167 (88.4)	189 (91.7)	0.260	178 (89.4)	178 (90.8)	0.648
Positive	22 (11.6)	17 (8.3)		21 (10.6)	18 (9.2)	
Tumor location						
Ascending colon	75 (39.7)	79 (38.3)	0.875	92 (46.2)	62 (31.6	0.039*
Transverse colon	11 (5.8)	16 (7.8)		12 (6.0)	15 (7.7)	
Descending colon	27 (14.2)	24 (11.7)		21 (10.6)	30 (15.3)	
Rectosigmoid	69 (36.5)	79 (38.3)		69 (34.7)	79 (40.3)	
Total colon	7 (3.7)	8 (3.9)		5 (2.5)	10 (5.1)	
Primary tumor size, cm						
<4	64 (33.9)	56 (27.2)	0.041*	54 (27.1)	66 (33.7)	0.294
4–6	77 (40.7)	110 (53.4)		96 (48.2)	91 (46.4)	
>6	48 (25.4)	40 (19.4)		49 (24.6)	39 (19.9)	
Histologic grade						
G1	56 (29.6)	8 (3.9)	<0.001***	25 (12.5)	39 (19.9)	<0.001***
G2	105 (55.6)	138 (67.0)		114 (57.3)	129 (65.8)	
G3	28 (14.8)	60 (29.1)		60 (30.2)	28 (14.3)	
Vascular invasion						
Negative	168 (88.9)	172 (83.5)	0.122	161 (80.9)	179 (91.3)	0.003**
Positive	21 (11.1)	34 (16.5)		38 (19.1)	17 (8.7)	
Nerve invasion						
Negative	162 (85.7)	162 (78.6)	0.067	158 (79.4)	166 (84.7)	0.170
Positive	27 (14.3)	44 (21.4)		41 (20.6)	30 (15.3)	
Dukes stage						
Stage A–B	132 (69.8)	112 (54.4)	0.002**	112 (56.3)	132 (67.3)	0.024*
Stage C–D	57 (30.2)	94 (45.6)		87 (43.7)	64 (32.7)	
Chemotherapy						
No	86 (45.5)	71 (34.5)	0.063	77 (38.7)	80 (40.8)	0.908
Yes	99 (52.4)	127 (61.7)		116 (58.3)	110 (56.1)	
Unknown	4 (2.1)	8 (3.9)		6 (3.0)	6 (3.1)	

*p*-values were calculated by comparing the expression of SNEP1 and SuFu with different clinical variables, respectively, using a chi-square test. *p* < 0.05 was considered statistically significant.

*p < 0.05, **p < 0.01, ***p < 0.001.

To investigate the significance of SNEP1 and SuFu protein expression levels concerning the clinical prognosis of CRC, we analyzed overall survival (OS) and disease-free survival (DFS) rates by Kaplan–Meier analysis and log-rank tests. We found that patients with higher SNEP1 protein levels exhibited much lower OS rates and DFS rates (Figs. 6D and S6F). Lower levels of SuFu contributed to shorter overall and disease-free survival (Fig. 6E and S6G). Finally, our multivariate survival analysis of the expression of these genes in CRC revealed that SNEP1 and SuFu, as well as Dukes stage, are significantly independent parameters for the prediction of CRC prognosis (DFS and OS) (Table 2). Taken together, these results reveal a critical role of SNEP1 in CRC progression and indicate that SNEP1 may be considered an independent prognostic biomarker for CRC.

**Table 2:** Univariate and multivariate analysis of factors associated with survival and recurrence of CRC patients.

	Overall survival								Disease-free survival							
	Univariate analysis				Multivariate analysis				Univariate analysis				Multivariate analysis			
	HR	95% CI		*p*-value	HR	95% CI		*p-*value	HR	95% CI		*p*-value	HR	95% CI		*p-*value
		Lower	Upper			Lower	Upper			Lower	Upper			Lower	Upper	
Sex (male versus female)	0.912	0.689	1.208	0.521					0.969	0.740	1.269	0.821				
Age (<60 versus ≥60)	1.165	0.881	1.540	0.285					1.105	0.845	1.445	0.464				
CEA (≤5 versus >5 ng/ml)	1.499	1.101	2.041	0.010	1.397	0.958	2.038	0.083	1.478	1.096	1.993	0.010	1.462	1.006	2.125	**0.046**
CA-199 (≤ 27 versus >27 U/ml)	1.805	1.279	2.547	0.001	1.154	0.765	1.740	0.495	1.756	1.252	2.462	0.001	1.178	0.773	1.793	0.446
CA-125 (≤34 versus >34 U/ml)	2.007	1.302	3.095	0.002	1.404	0.868	2.273	0.167	1.889	1.237	2.883	0.003	1.573	0.954	2.591	0.076
General type (bulge type versus ulcer type)	1.635	1.174	2.277	0.004	1.214	0.807	1.827	0.352	1.735	1.257	2.396	0.001	1.388	0.919	2.098	0.119
Tumor location (colon versus rectosigmoid)	0.899	0.673	1.200	0.470					0.881	0.667	1.164	0.374				
Primary tumor size (≤5 versus >5 cm)	0.758	0.573	1.003	0.052					0.733	0.560	0.959	0.023	0.696	0.493	0.981	0.039
Histologic Grade (grade 1–2 versus grade3)	1.624	1.181	2.235	0.003	1.256	0.816	1.933	0.300	1.480	1.084	2.019	0.014	1.215	0.766	1.927	0.408
Vascular invasion (negative versus positive)	2.460	1.725	3.509	6.671E-07	1.354	0.861	2.130	0.189	2.124	1.494	3.019	2.696E-05	1.190	0.730	1.940	0.485
Nerve invasion (negative versus positive)	1.806	1.291	2.525	0.001	1.507	0.996	2.282	0.053	1.640	1.180	2.279	0.003	1.330	0.881	2.007	0.175
History of intestinal polyps (negative versus positive)	0.878	0.541	1.425	0.597					0.821	0.513	1.316	0.413				
Dukes stage (A–B versus C–D)	3.030	2.290	4.009	8.557E-15	1.838	1.273	2.656	0.001**	2.803	2.142	3.668	5.853E-14	1.650	1.129	2.413	0.010*
Chemotherapy (yes versus no)	0.744	0.553	1.000	0.050					0.643	0.482	0.857	0.003	0.734	0.496	1.084	0.120
SNEP1 expression (low versus high)	1.694	1.275	2.250	2.733E-04	1.778	1.240	2.548	0.002**	1.586	1.208	2.081	0.001	1.683	1.175	2.410	0.004**
SuFu expression (low versus high)	0.643	0.486	0.852	0.002	0.589	0.410	0.844	0.004**	0.662	0.505	0.867	0.003	0.592	0.414	0.845	0.004**

All data are the number of patients (%). *p < 0.05, **p < 0.01.

### SNEP1 induces vismodegib-resistance in colorectal cancer cells

**Fig. 7:**
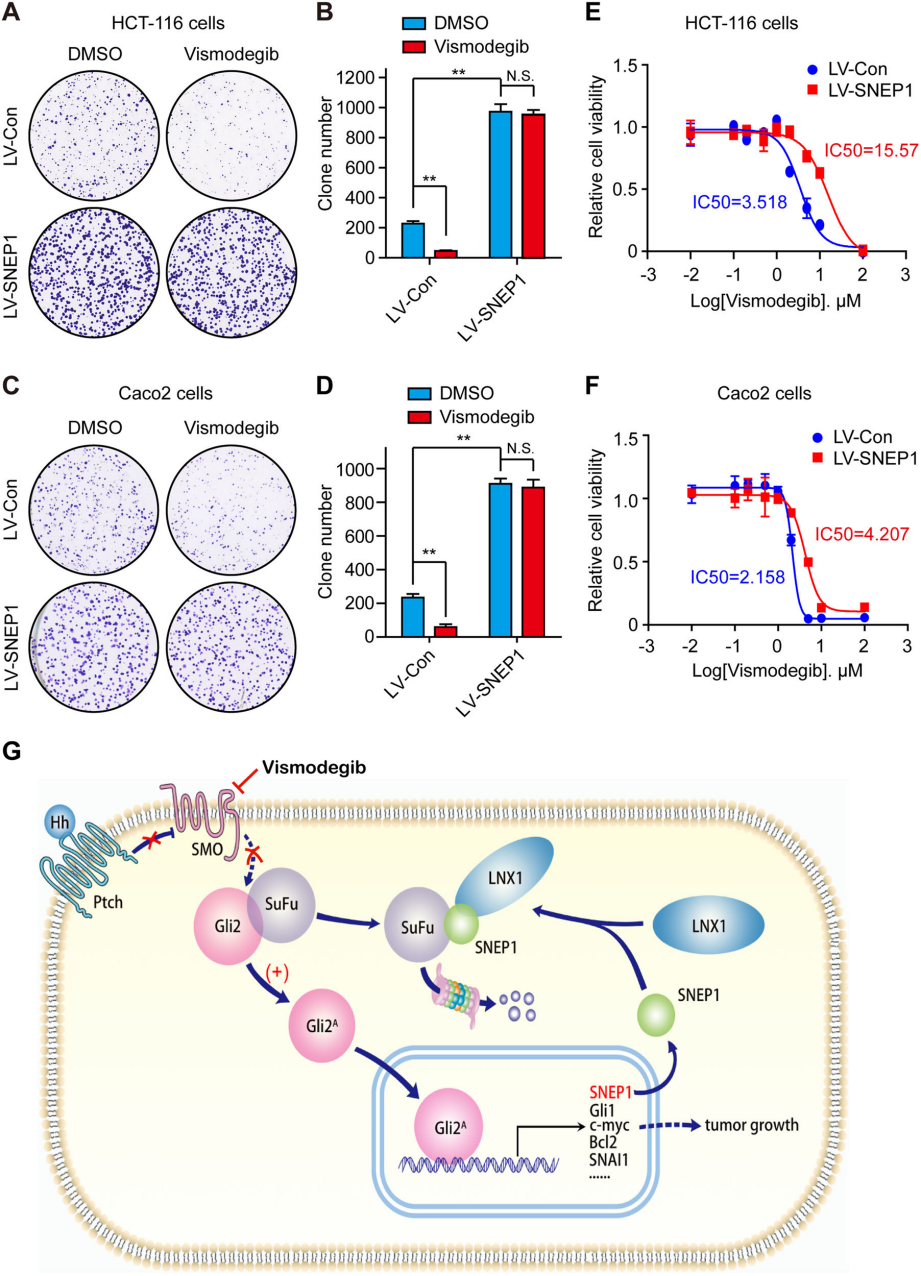
SNEP1 induces vismodegib-resistance in colorectal cancer cells. **A–D** HCT-116 or Caco2 cells stably expressing LV-SNEP1 were seeded in a 6-well plate for 14 days, and treated with 2.5 μM vismodegib. The cells were then stained with 0.5% crystal violet (w/v) (**A** and **C**). Quantitative analysis was performed using Image J software (**B** and **D**). The bar graph displays the mean ± SD, *n* = 3, **p < 0.01, N.S., not significance. **E**, **F** Relative cell viability and IC50 of vismodegib in control or SNEP1-overexpressing HCT-116 cells (**E**), Caco2 cells (**F**). Data represent mean ± SD of three separate experiments. **G** Schematic showing how SNEP1 and LNX1 work together to degrade SuFu via a ubiquitin-dependent mechanism to promote CRC development by activating the Hh pathway.

Vismodegib (GDC-0449), a Smo-targeting inhibitor, was recently approved by FDA for the treatment of basal cell carcinoma. Vismodegib has preclinical activity in colorectal cancer (CRC) models, but it failed to show benefit in Phase II clinical of CRC^40^. Activation of Smo-independent Hh signaling may induces vismodegib-resistance. We found SNEP1 activates the Hh signaling pathway by regulating SuFu, which is Smo downstream component. We reasoned that elevated expression of SNEP1 may induce resistance to vismodegib in CRC. To evaluate this conjecture, clone formation and cell viability assay were used to analyze the effects of vismodegib in control and SNEP1-overexpressing colorectal cancer cells. Overexpression of SNEP1 suppressed the inhibitory effects of vismodegib on clone formation (Fig. 7A–D). Moreover, the IC50 of vismodegib is increased in SNEP1-overexpressing colorectal cancer cells (Fig. 7E, F). Taken together, these results indicate that overexpression of SNEP1 reduced the sensitivity to vismodegib in CRC cell lines, which has clinical implications for investigations of Hh inhibitors resistance in CRC therapy.

## Discussion

Hh signaling promotes the expression of a series of oncogenes by activating the Gli transcriptional factors that bind to the consensus sequence GACCACCCA through their zinc finger domains^41^. Among the three Gli homologs, Gli2 plays a more critical role in development and tumorigenesis^42,43^, and deficiency of Gli2, but not Gli1, in mice leads to embryonic lethality at later embryonic stages^44^. Here, we report SNEP1 as a novel target of the Gli transcription factors in response to Hh signaling. Our data indicate that SNEP1 acts as a positive feedback regulator of Hh signaling. As an Hh target, SNEP1 binds to LNX1 and enhances its E3 ubiquitin ligase activity toward SuFu, thereby mediating SuFu proteasomal degradation in response to Hh signaling. As a result, Gli2 becomes activated, enters the nucleus and induces the expression of its target genes to promote cell proliferation and survival. It is through this positive feedback regulation of Hh signaling that SNEP1 acts as an oncoprotein to promote CRC development. Interestingly, although Hh signaling is a conserved signaling pathway from *Drosophila* to humans, SNEP1 is a novel gene found in humans, with no homologs even in many mammals, including mice. This indicates that this unique Hh regulator might play certain roles in human development and tumors, but these roles remain to be discovered in the future.

Two E3 ligase complexes have been previously reported to mediate SuFu ubiquitination in mammals. Intriguingly, these two E3 ligases have opposite effects on SuFu stability: SCF^Fbxl17^-mediated ubiquitination facilitates SuFu degradation^28^, whereas β-arrestin2-Itch–mediated K63 ubiquitination protects SuFu from proteolysis^29^. We identified LNX1 as another E3 ligase that promotes SuFu ubiquitination and degradation. LNX1 and SCF^Fbxl17^ may utilize different mechanisms to target SuFu for degradation. Indeed, they target different lysine residues in the SuFu protein, as LNX1 ubiquitinates SuFu at K59 and K470, and SCF^Fbxl17^ acts at K257, while Itch ubiquitinates at K321 and K457. It would be interesting to explore whether there is any interplay between SCF^Fbxl17^ and the SNEP1-LNX1 complex in the regulation of SuFu stability in response to Hh signaling or other signals and whether β-arrestin2-Itch competes with the SNEP1-LNX1 complex to regulate SuFu stability.

The reduction in SuFu protein levels in response to Hh signaling has been known for more than a decade, and many regulators of this process, including protein kinases, interacting adaptors, and E3 ligases, have been reported. In *Drosophila*, Hh-induced BTB protein expression downregulates SuFu levels by repressing its translation without affecting its stability^45,46^. It has also been shown that dual phosphorylation of SuFu by PKA and GSK3β leads to SuFu stabilization^30^, while dephosphorylated SuFu is recognized by Fbxl17 for Skp1-mediated proteolysis^28^. Bcl2, an Hh target, was also reported to directly bind to SuFu and facilitate its turnover, consequently suppressing SuFu interaction with Gli proteins^47^. Our studies, as presented here, uncover SNEP1 as novel Hh target was also directly bind to SuFu and facilitate its degradation, consequently activating Hh signaling pathway.

It has been shown that aberrant Hh signaling plays a key role in the initiation and/or maintenance of gastrointestinal tumors, including CRC^13,14,48^. Three principal models of aberrant Hh signaling have been proposed in Hh pathway-dependent cancer: (i) ligand-independent signaling, (ii) ligand-dependent autocrine signaling, and (iii) ligand-dependent paracrine signaling. Ligand-independent Hh signaling is associated with a subset of human tumors; this type includes PTCH1 inactivating mutations, Smo activating mutations, SuFu loss-of-function mutations, and gene amplifications of Gli1^49^. As SNEP1 expression in the intestinal epithelium activated Hh signaling by facilitating SuFu degradation and consequently promoted CRC cell proliferation and growth in vitro and *vivo*, we speculated that SNEP1 might have a positive effect on CRC progression by activating Hh signaling in a ligand-independent manner. Indeed, ectopic expression of SNEP1 in mice increased the number and size of tumors in AOM-DSS-induced CRC. Moreover, our analyses of a cohort of 395 pairs of primary CRC tissues and patients suggested that SNEP1 may be used as an independent prognostic factor for CRC.

Smoothened (Smo), a vital receptor of Hh signaling pathway, is an important therapeutic target in Hh signaling pathway-related cancer therapy. Vismodegib (GDC-0449) is the most widely used drugs for targeting the Hh pathway, the first drug approved by the FDA to treat basal cell carcinoma (BCC). However, Vismodegib appear to be largely ineffective in the treatment of solid tumors other than BCC^40^. In this study, we demonstrates that SNEP1 activates Hh signaling by regulating downstream of the Smo, and thereby induces resistance to Smo inhibitors in CRC cells. Moreover, we found that Gli inhibitor GANT61 attenuates SNEP1-induced Hh signaling activation and CRC cell proliferation. These findings suggest that SNEP1 may cause the failure of clinical treatment of Smo inhibitor in CRC.

In summary, our comprehensive basic and clinical studies, as described here, unravel two new players, SNEP1 and LNX1, in the Hh pathway. Biochemically, SNEP1 can partner with LNX1, a Ring E3 ligase, to ubiquitinate and degrade SuFu, a suppressor of the Hh responsive Gli2 transcriptional factor. Since SNEP1 is also an authentic transcriptional target of Gli2, this LNX1 coworker acts as a positive feedback regulator of Hh signaling. Biologically, highly expressed SNEP1, such as in xenograft or primary human CRC tumors, can promote CRC cell proliferation and survival as well tumorigenesis by inhibiting SuFu and consequently activating the Hh pathway. Clinically, our analysis of a large cohort of primary CRC tissues and patients suggests that SNEP1 could serve as a prognostic biomarker for CRC and might be used as a target for future anti-CRC therapy development.

## Materials and methods

### Cell lines and transfection

Transformed human embryonic kidney cell line HEK-293T and human CRC cell lines, including HT-29, HCT-116, Caco2, and SW620, were purchased from ATCC. All cell lines were authenticated using short tandem repeat profiling and were negative for mycoplasma contamination detected via PCR-based assay. The cells were cultured in Dulbecco’s modified Eagle’s medium (Gibco) supplemented with 10% FBS (Gibco) and antibiotics (100 U/ml streptomycin and 100 μg/ml penicillin, Invitrogen). Cells were transiently transfected with Lipofectamine 3000 (Invitrogen, Carlsbad, CA, USA) in accordance with the manufacturer’s instructions.

### Antibodies, reagents, and constructs

SNEP1 polyclonal antibody was purified from rabbit by Beijing Boer Mai Biotechnology Company, China. Other antibodies were purchased from Abcam (Gli2, ab26056; SuFu, ab52913; Ptch1, ab55629), Cell Signaling Technology (Bcl2, 2876; HA, 3724s), Sigma (Flag [M2], F3165; c-Myc, M4439; GFP, G1544), Santa Cruz (Ki67, sc-1540; PARP-1, sc-7150; Ub, sc-8017; FoxM1, sc-376471), LSBio (LNX1, LS-C175459), Millipore (GAPDH, MAB374), Genetex (Sox2, GTX101506) and Thermo Fisher Scientific (goat anti-rabbit IgG, 31460; goat anti-mouse IgG, 31430).

Reagents were purchased from Selleck (Vismodegib, S1082), Invitrogen (Lipofectamine 3000, L3000015; ECL, WP20005), Solarbio (Doxycycline, SD8430) and Sigma (GANT61, G9048), Wako (Sodium Dextran Sulfate, 196-13401). The other analytical-grade chemicals were purchased from Sigma-Aldrich (St. Louis, MO, USA).

Expression plasmids of human full-length *Gli2* (Cat. RC217291) were purchased from OriGene Technologies (Rockville, MD). The human full-length *SNEP1* (NM_001012716) construct was subcloned into pcDNA3.1-Flag (Invitrogen). The shRNAi-Gli2 expression vectors were purchased from GeneChem (Shanghai, China). The shRNAi-SNEP1 and shRNAi-LNX1 expression vectors were generated using the BLOCK-iT™ Pol II miRNAi Expression Vector Kit (K4936-00, Invitrogen, Carlsbad, CA, USA) in accordance with the manufacturer’s protocol. The target sequences of the aforementioned shRNAi expression constructs are listed in Appendix Table S1. All stably transfected cell lines were treated with 1 μg/ml puromycin, and selected clones were identified by immunoblotting.

### Hh target gene screening

HT-29 cells infected by lentivirus expressing Gli2 or empty vector or treated with GANT61 or DMSO were harvested for RNA extraction with TRIzol reagent (Invitrogen). Gene expression profiles were determined via next-generation sequencing (NGS) with Illumina NovaSeq by Novogene Co., Ltd. (Beijing, China), and genes with expression changes over 2-fold and adjusted *p*-values < 0.05 were considered differentially expressed genes (DEGs). All raw data are available at the sequence read archive (SRA) with accession no. PRJNA623247. To screen the novel target genes with unknown functions, the Gene Ontology database was used to annotate the DEGs, and these genes that had no annotation except that inferred from physical interaction (IPI) were identified as function unknown.

### Immunoblot (IB) and quantitative polymerase chain reaction (qPCR)

Cells were harvested and lysed in lysis buffer (0.5% Lubrol-PX, 50 mM KCl, 2 mM CaCl2, 20% glycerol, 50 mM Tris-HCl, and inhibitors of proteases and phosphatases, pH 7.4). Cell lysates were cleared of debris via centrifugation at 4 °C and 12,000 rpm for 15 min and subjected to IB with the indicated antibodies, as described previously 50. Total RNA was harvested using TRIzol Reagent (Life Technologies™) and evaluated via qPCR. Briefly, total RNA (1 μg) was employed to prepare cDNA via reverse transcription using a PrimeScript® RT reagent Kit with gDNA Eraser (Takara, DRR047A). qPCR was carried out in an ABI StepOnePlus™ Real-Time PCR System (Applied Biosystems) using SYBR® Premix Ex Taq™ Tli RnaseH Plus (Takara, DRR820A). The primers used are shown in Appendix Table S2. Data are represented as the mean ± SD from at least three independent experiments.

### Yeast two-hybrid screening

SNEP1 was subcloned into the pGBKT7 vector as bait, resulting in a fusion with the Gal4 DNA-binding domain. The resulting plasmid and Saccharomyces cerevisiae strain Y187 containing the cDNA library were simultaneously transformed into the yeast Y2HGold strain, as described previously 48.

### Immunoprecipitation and GST pull-down assay

HEK-293T cells were transfected with GFP-SNEP1 and Flag-SuFu or Flag-LNX1 for 48 h and harvested for immunoprecipitation analysis, as described previously 48. For the GST pull-down assay, GST-tagged SNEP1 was expressed in *E. coli* and conjugated to glutathione-Sepharose 4B beads (Sigma-Aldrich). Protein–protein interaction assays were processed using cell lysates with mammalian-expressed HA-SuFu or Flag-LNX1. The cell lysates were incubated with the indicated GST fusion proteins before being immobilized on glutathione-Sepharose beads. Bound proteins were eluted from the beads and analyzed via sodium dodecyl sulfate polyacrylamide gel electrophoresis/Coomassie Blue staining and/or subjected to immunoblotting.

### Chromatin immunoprecipitation (ChIP) assay

HT-29 cells at 90% confluence were cross-linked in 1% formaldehyde. Their DNA was sonicated into fragments of 100 to 400 bp using a Bioruptor Sonicator (Diagenode) for 30 cycles of 1 s on with 2 s off. The lysates were precleared in BSA-blocked protein A/G beads and incubated with specific antibodies or IgG control overnight. After washing, DNA was eluted and reverse cross-linked at 65 °C overnight. Eluted DNA was used as a template for semiquantitative PCR. The input control was obtained from the supernatant before precipitation. The predictive binding sequences and the primers used for SNEP1 promoters are listed in Appendix Table S3.

### Construction of luciferase reporter vectors and luciferase assay

In silico analysis of transcription factor Gli2-binding sites in the human SNEP1 5ʹ-upstream region (−1062 to +971) was performed by using Genomatix MatInspector software (http://www.genomatix.de/). To construct the reporter vector for the luciferase assay, the 5ʹ-fragment of human SNEP1 containing Gli2-binding sites was amplified via PCR and cloned into the firefly luciferase reporter plasmid pGL3-Enhancer (Promega, Madison, WI, USA). Cloned promoter sequences were validated via Sanger DNA sequencing. The primers used for the luciferase reporter constructs are listed in Appendix Table S4. For the luciferase reporter assays, the pGL3-Enhancer-Luc reporter plasmids (0.2 μg/well) and the internal control plasmid pRL-TK (5 ng/well) were transfected into HEK-293T cells. The constructs of Myc-Gli2, shRNAi-Gli2, or empty vector (0.4 μg/well) were cotransfected for 24 h, and reporter gene activity was assayed using the Dual Luciferase Assay System (Promega) in accordance with the manufacturer’s protocol. All experiments comprised three biological replicates.

### Colony formation assay

For the colony formation assay, CRC cells stably infected with LV-SNEP1 or LV-shSNEP1 were seeded into 6-well plates. Two weeks later, the cells were fixed and stained with 0.5% crystal violet (w/v). The number of colonies was enumerated microscopically and determined via ImageJ software (National Institutes of Health, Bethesda, MD, USA).

### Lentiviral infection and mouse model

For lentiviral infection, HT-29, HCT-116, SW620, and CaCo2 cells, at a density of 4 × 10^5^, were incubated with 1 × 10^8^ IU of virus and 8 μg/ml of polybrene (Sigma-Aldrich, St. Louis, MO, USA) for 12 h. Protein expression was induced in 1 μg/ml doxycycline (Sangon Biotech, Shanghai, China) for 48 h.

For ex vivo experiments, 2 × 10^7^ stably infected HT-29 (LV-sh-control and LV-sh-SNEP1) and HCT-116 (LV-control and LV-SNEP1) cells were resuspended in sterilized PBS (200 μl) and hypodermically injected into bilateral inguinal parts of 5-week-old female BALB/c-nu mice (SLAC Laboratory Animal CO., Ltd., Hunan, China). Three days after injection, mice were administered 2 mg/ml doxycycline in drinking water, which was replenished every 2 days. Tumor sizes on both sides of mice were monitored using a Vernier caliper every alternate day. After 2 weeks, xenografts were harvested for IHC and IB analysis. Eight female nude mice were used for each group.

CAG-loxP-Stop-loxP-SNEP1 cds-IRES-EGFP-WPRE-pA was knocked in the Rosa26 site to generate B6-Gt(ROSA)26Sor^em1(CAG-LSL-SNEP1-IRES-eGFP)/NCU^ (LSL-SNEP1) mice, B6-Tg^(Vil1-cre)997Gum/J^ (vil-cre) mice were purchased from Shanghai Model Organisms Center. LSL-SNEP1/+ mice were crossed with vil-cre/+ mice to generate LSL-SNEP1/+; vil-cre/+ mice and mice lacking vil-cre were used as controls.

For the inflammation-dependent colorectal cancer model, mice were injected with AOM (Sigma-Aldrich, A5486) at 10 mg per kg bodyweight before oral administration of 2.5% DSS solution (5 ml/day) for 5 days. DSS cycles were repeated three times within 14 days of normal drinking water (Fig. 2I). The DSS solution was renewed after 2 days. For the genetic colorectal cancer model, mice were kept normally until 16 weeks for sacrifice. This study was approved by the Ethics Committee of the Frist Affiliated Hospital of Nanchang University (Nanchang, China).

### Patients and clinical samples

A cohort of 395 CRC patients undergoing surgery was reviewed at the First Affiliated Hospital of Nanchang University between January 2009 and August 2014. All patients had primary tumors that were untreated before surgery. Biopsy specimens were reviewed by a pathologist to ensure that all the specimens contained tumors and adjacent normal tissues. We defined tumor size as the maximum tumor diameter measured intraoperatively in the tumor specimens. Histological types of the 395 samples were defined in accordance with the WHO classification criteria as grade I (64 cases), grade II (243 cases), and grade III (88 cases). Clinical stage was defined in accordance with the 7th edition of the AJCC. This study was approved by the Ethics Committee of the Frist Affiliated Hospital of Nanchang University (Nanchang, China).

### Immunohistochemical (IHC) analysis

Paraffin sections (3 μm thick) of formalin-fixed CRC tissue and adjacent tissue were dewaxed, rehydrated, and incubated in 3% hydrogen peroxide solution for 10 min. Antigen retrieval was performed by heating them in EDTA buffer (pH 9.0) for 24 min and cooling them down naturally. Normal goat serum (10%) was used to block nonspecific staining, and then the tissue sections were exposed to the indicated antibodies. The stained sections were observed by at least two independent investigators blinded to the histopathological features of the samples. The German semiquantitative scoring system was employed for assessing the staining intensity and stained area. Each specimen was assigned a score in accordance with the staining intensity of the nucleus or cytoplasm (no staining, not detected = 0; weak staining, light yellow = 1; moderate staining, yellowish brown = 2; strong staining, brown = 3) and the extent of staining in cells (0% = 0, 1–24% = 1, 25-49% = 2, 50–74% = 3, 75–100% = 4). The final immunoreactive score was determined by multiplying the intensity score with the score of the extent of staining, ranging from 0 (the minimum score) to 12 (the maximum score).

### Statistical analysis

Differences in quantitative data between two groups were analyzed using two-sided paired or unpaired Student’s *t*-tests. IHC scores between two or three independent groups were compared using the Mann–Whitney *U*-test or the Kruskal–Wallis H-test. The *χ*² test was used to analyze the correlation between gene expression and clinicopathological characteristics. The Kaplan–Meier method and the log-rank test were performed for survival analysis. The Cox proportional hazards model was used to determine independent factors influencing survival and recurrence based on the variables selected from the univariate analysis. The sample size were calculated by G Power 3.1. A *p*-value < 0.05 was considered statistically significant. Statistical analyses were performed using SPSS software version 21.0 (SPSS, Chicago, IL, USA).

## Supplementary information

Supplementary Figure Legends

Supplementary Figure 1

Supplementary Figure 2

Supplementary Figure 3

Supplementary Figure 4

Supplementary Figure 5

Supplementary Figure 6

Supplementary Table

## Data Availability

All raw RNA-Seq data are available at the sequence read archive (SRA) with accession no. PRJNA623247.
