# Supplementary material for: Positive feedback of SuFu negating protein 1 on Hedgehog signaling promotes colorectal tumor growth
Source: Cell Death Dis. 2021 Feb 19;12(2):199. doi: 10.1038/s41419-021-03487-0 (PMC7896051; doi:10.1038/s41419-021-03487-0)
Supplement: Supplementary file 1 — Supplementary Figure Legends [file 41419_2021_3487_MOESM1_ESM.doc]

**Supplementary Figure Legends**

**Figure S1. Supplementary to Figure 1.**

**(A)** Gli2 knockdown reduces SNEP1 protein levels. Caco2 cells were transfected with shRNAi-Gli2 plasmids for 72 h and harvested for IB with indicated antibodies.

**(B)** Pharmacological repression of Gli2 reduces SNEP1 mRNA levels. Caco2 cells were treated with GANT61 for 48 h and harvested for RNA extract and qPCR. Data are presented as mean ± SD (n=3). **, *p* < 0.01.

**(C-D)** Antibody validation. The antibody recognizes both the endogenous (C) and exogenous (D) SNEP1.

**Figure S2. Supplementary to Figure 2.**

**(A)** The expression of SNEP1 in human colon carcinoma cell lines. Cells lysates extracted for Western analysis

**(B-C)** IHC analysis of the xenografts. Tumors were isolated, fixed, and subjected to IHC assays with indicated antibodies.

**(D)** Ectopic SNEP1 leads to increase of Gli2 protein levels, decrease of SuFu protein levels *ex vivo*. Two pairs of xenografts were analyzed via IB with indicated antibodies.

**(E)** SNEP1 knockdown leads to decrease of Gli2 protein levels, but increase of SuFu protein levels *ex vivo*. Two pairs of the xenografts were analyzed via IB with indicated antibodies.

**(F)** IHC analysis of the tumors. Tumors were isolated from DSS-induced CRC models, fixed, and subjected to IHC assays with the indicated antibodies.

**(G)** Ectopic SNEP1 leads to decrease of SuFu protein levels, increase of Gli2 protein levels *in vivo*. Two pairs of CRC samples were analyzed via IB with indicated antibodies.

**Figure S3. Supplementary to Figure 3.**

**(A)** SNEP1 activates Hh signaling. HCT-116 cells with ectopic SNEP1 expression were harvested for IB.

**(B)** SNEP1 activates Hh signaling. HT-29 cells with stable SNEP1 knockdown were harvested for qPCR. Data are presented as mean ± SD (n=3). *, *p* < 0.05, **, *p* < 0.01.

**(C)** SNEP1 facilitates CRC cell proliferation dependent on activation of the Hh Pathway. HCT-116 cells were infected with LV-SNEP1 or control lentivirus, and treated with 2.5 μM GANT61. The cell numbers were counted each day. *, *p* < 0.05, **, *p* < 0.01.

**(D)** SNEP1 overexpression does not affect SuFu mRNA levels. HCT-116 cells stably expressing SNEP1 were cultured for 48 h and harvested for qPCR analysis. Data were shown as mean ± SD, n = 3, **, *p* < 0.05.

**(E-F)** Mapping of the SuFu binding domain of SNEP1 by a GST pull-down assay. The prokaryotic expressed GST-tagged SNEP1 or GST protein alone was incubated with lysates isolated from HEK-293T cells that harbored ectopic HA-SuFu fragments. Bound proteins were detected via IB analysis with anti-HA antibody.

**(G)** GST pull-down assay of SNEP1 with SuFu (110–174). The prokaryotic expressed SuFu (110–174) was incubated with HEK-293T cell lysates with overexpressed SNEP1. The protein complexes pulled down with GST were detected via IB.

**(H)** SNEP1 interacts with SuFu (sites 110–174). The prokaryotic expressed GST-SNEP1 or GST alone was incubated with lysates isolated from HEK-293T cells that overexpressed HA-SuFu WT or mutant (Δ110–174). Bound proteins were detected via IB with an anti-HA antibody.

**(I)** SuFu degradation was attenuated by the fragment of SuFu (110-174). Cycloheximide (CHX) (100 μg/ml) was incubated for the indicated period with HEK-293T cells transfected with the fragment of SuFu (110-174). Cell lysates were harvested for IB with anti-SuFu antibody.

**(J)** Quantitative analysis of SuFu protein levels shown in (I), using ImageJ software.

**Figure S4. Supplementary to Figure 4.**

**(A-D)** Mapping of the LNX1-binding domain on SNEP1. LNX1 contains four PDZ domain (A), one of which mediated its interaction with SNEP1. Mapping of the LNX1-binding domain on SNEP1 via a co-IP assay (B) or GST pull-down assays (C and D). In GST pull-down assays, the prokaryotic expressed GST-SNEP1 or GST alone was incubated with HEK-293T cell lysates that overexpressed Flag-LNX1 fragments. Bound proteins were detected via IB with an anti-Flag antibody or Coomassie Brilliant Blue staining.

**(E)** LNX1 overexpression reduces SuFu protein levels. HEK-293T cells were transfected with different doses of Flag-LNX1 construct for 48 h and harvested for IB analysis.

**(F-G)** Construction of LNX1 shRNA plasmids. HEK-293T cells were transfected with different shRNA constructs of LNX1 for 48 h, with ectopic LNX1 or not. Cell lysates were subjected to IB with anti-LNX1 and anti-SuFu antibody.

**(H-I)** SuFu degradation was attenuated by LNX1 depletion. (H) Cycloheximide (CHX) (100 μg/ml) was incubated for the indicated period with HEK-293T cells transfected with shRNA-LNX1. Cell lysates were harvested for IB with anti-SuFu antibody. (I) Quantitative analysis of SuFu protein levels shown in (H), using ImageJ software.

**(J)** Ectopic SNEP1 reduces SuFu protein levels. IB analysis of HEK-293T cell lysates that expressed Flag-LNX1 with or without GFP-SNEP1.

**(K)** SuFu (Δ110-174) resists to LNX1 mediated degradation. IB analysis of HEK-293T cell lysates that expressed Flag-LNX1 with wide type SuFu or mutated SuFu (Δ110-174).

**(L)** Ectopic Gli2 increases SuFu-LNX1 interaction which dependent on SNEP1. HCT-116 cells were cotransfected with myc-Gli2 with or without sh-SNEP1 plasmids and treated with MG132 overnight before harvesting. Cell lysates were subjected to a Co-IP assay.

**Figure S5. Supplementary to Figure 5.**

**(A)** LNX1 knockdown drastically reduces SuFu ubiquitination. HEK-293T cells were transfected with shRNA control or shLNX1 for 48 h and treated with MG132 overnight before harvest. Cell lysates were subjected to IP with anti-SuFu antibody and protein A/G beads overnight. Polyubiquitin chains bound to SuFu were assessed via IB.

**(B)** Recombinant SNEP1, SuFu, and LNX1 from *E. coli*. GST-SuFu, GST-LNX1 (1-600), and SNEP1 were expressed in and purified from *E. coli*. Purified proteins were analyzed via sodium dodecyl sulfate polyacrylamide gel electrophoresis (SDS-PAGE), followed by Coomassie Brilliant Blue staining.

**(C)** Schema of lysines in the SuFu protein sequence. All lysines in SuFu protein are shown, and the conserved lysine residues are shown in red.

**(D)** SuFu (K59R) and SuFu (K470R) were partially resistant to LNX1-mediated degradation. HEK-293T cells were transfected with GFP-SuFu, GFP-SuFu (K59R), GFP-SuFu (K398R), GFP-SuFu (K467R), or GFP-SuFu (K470R) and Flag-vector or Flag-LNX1 for 48 h before harvest. Cell lysates were assessed via IB.

**(E)** SuFu (K59/470R) was completely resistant to LNX1-mediated degradation. HEK-293T cells were transfected with GFP-SuFu or GFP-SuFu (K59/470R) and Flag-vector or Flag-LNX1 for 48 h before harvest. Cell lysates were assessed via IB.

**(F)** SuFu-K59/470 was resistant to LNX1-mediated degradation in a protein half life assay. HEK-293T cells transfected with GFP-SuFu or GFP-SuFu (K59/470R) and Flag-LNX1, respectively were incubated for different time points with CHX (100 μg/ml). Cell lysates were assessed via IB with indicated antibodies. SE, short exposure; LE, long exposure.

**Figure S6. Supplementary to Figure 6.**

**(A)** Representative images of IHC staining of SuFu and SNEP1 expression in two serial sections of the same tumor of different histological grades (1–3).

**(B-C)** Correlation of SNEP1 (B) and SuFu (C) expression with pathological grades. SNEP1 and SuFu expression scores are shown as box plots. Sample numbers of different clinical stages are described below for each respective group. Data were analyzed using the Kruskal–Wallis test. In (B) and (C), horizontal lines represent the median; the bottom and top of the boxes represent the 25th and 75th percentiles, respectively; and the vertical bars represent the range of data. Outliers are marked with a circle.

**(D)** Western blotting analysis of SNEP1, SuFu and LNX1 expression in twelve pairs of randomly selected CRC and matched adjacent non-tumor tissues. C, carcinoma tissue; N, matched adjacent non-tumor tissue.

**(E)** Correlation between SNEP1, LNX1 and SuFu relative expression levels in CRC. Quantitative analysis of protein levels shown in (D), protein levels were relative to β-actin.

**(F-G)** Kaplan–Meier estimates of disease-free survival of CRC patients between the negative/low and medium/high expression groups for SNEP1 (F) and SuFu (G). Marks on graph lines represent censored samples. P value refers to two-sided log-rank tests.
