# Supplementary figures and images for: Positive feedback of SuFu negating protein 1 on Hedgehog signaling promotes colorectal tumor growth
Source: Cell Death Dis. 2021 Feb 19;12(2):199. doi: 10.1038/s41419-021-03487-0 (PMC7896051; doi:10.1038/s41419-021-03487-0)

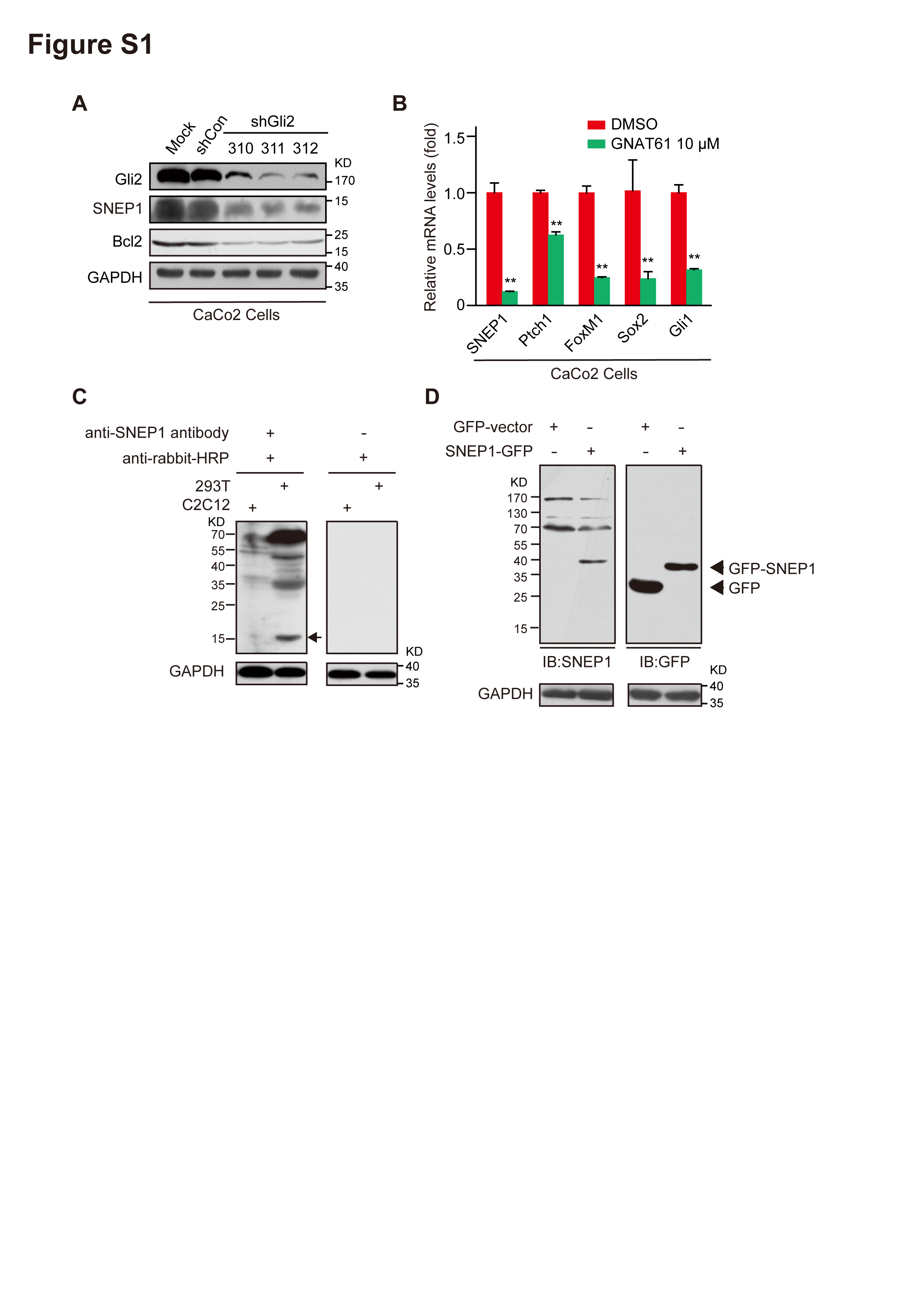

Supplement: Supplementary file 2 — Supplementary Figure 1 [file 41419_2021_3487_MOESM2_ESM.tif]

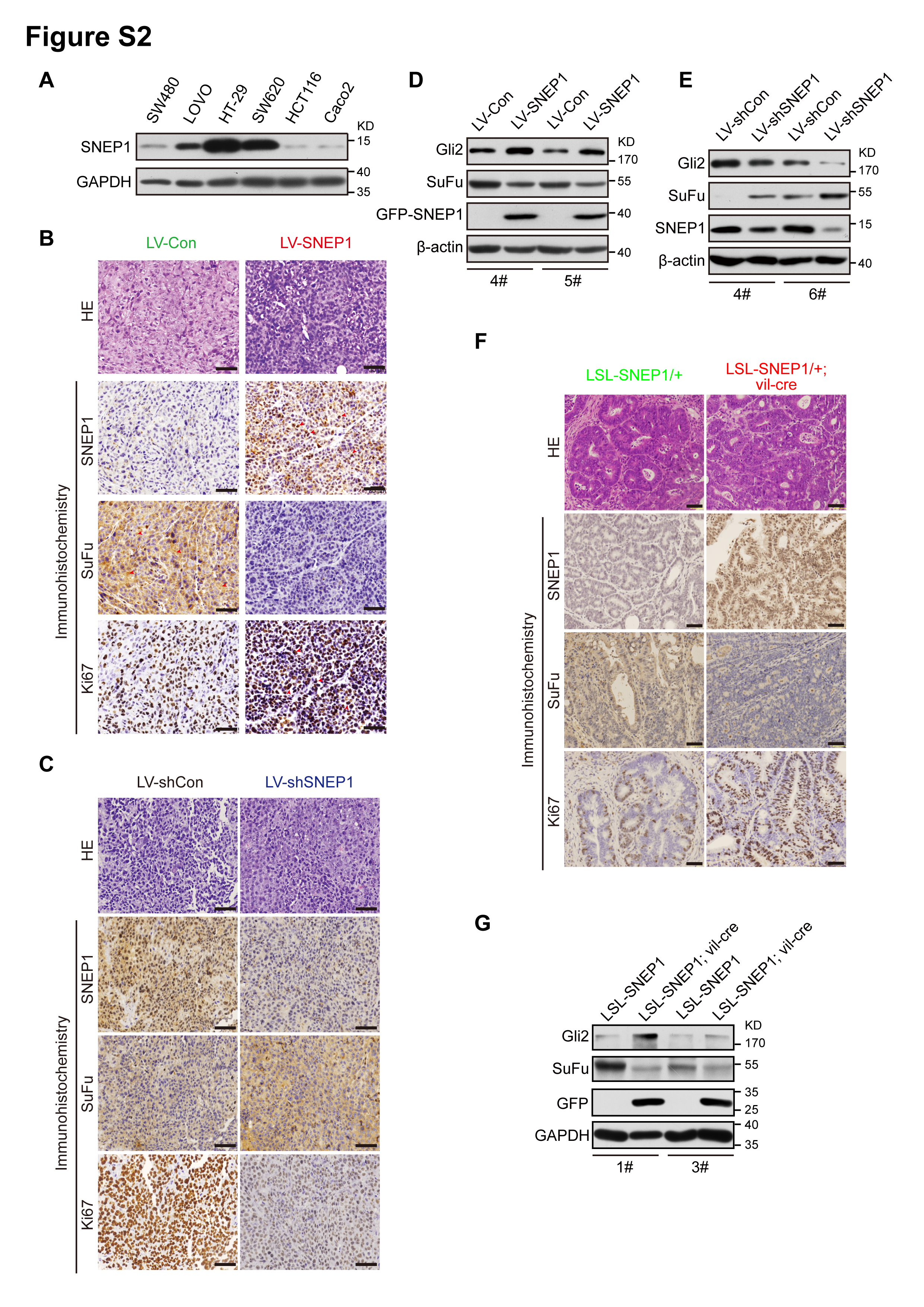

Supplement: Supplementary file 3 — Supplementary Figure 2 [file 41419_2021_3487_MOESM3_ESM.tif]

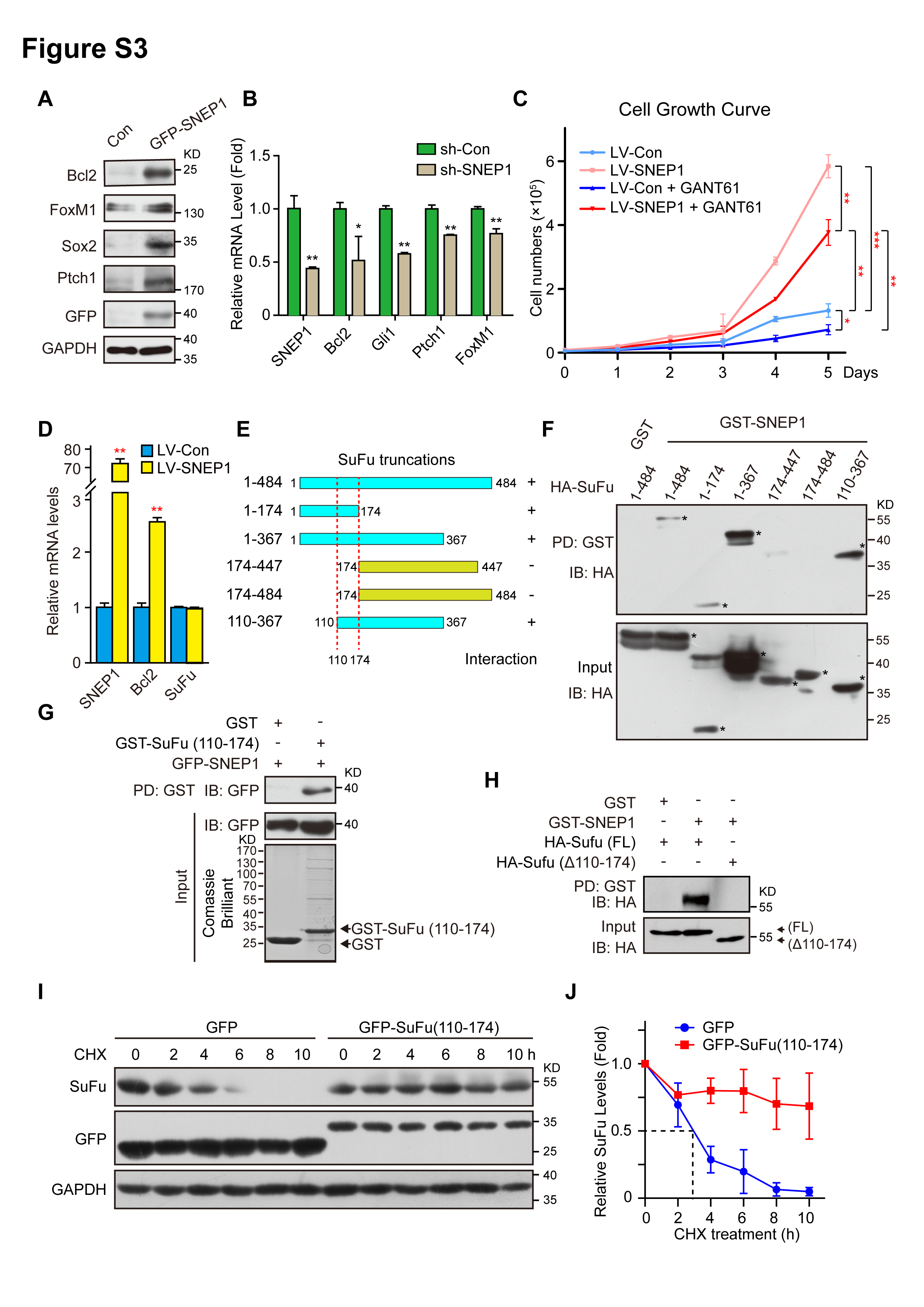

Supplement: Supplementary file 4 — Supplementary Figure 3 [file 41419_2021_3487_MOESM4_ESM.tif]

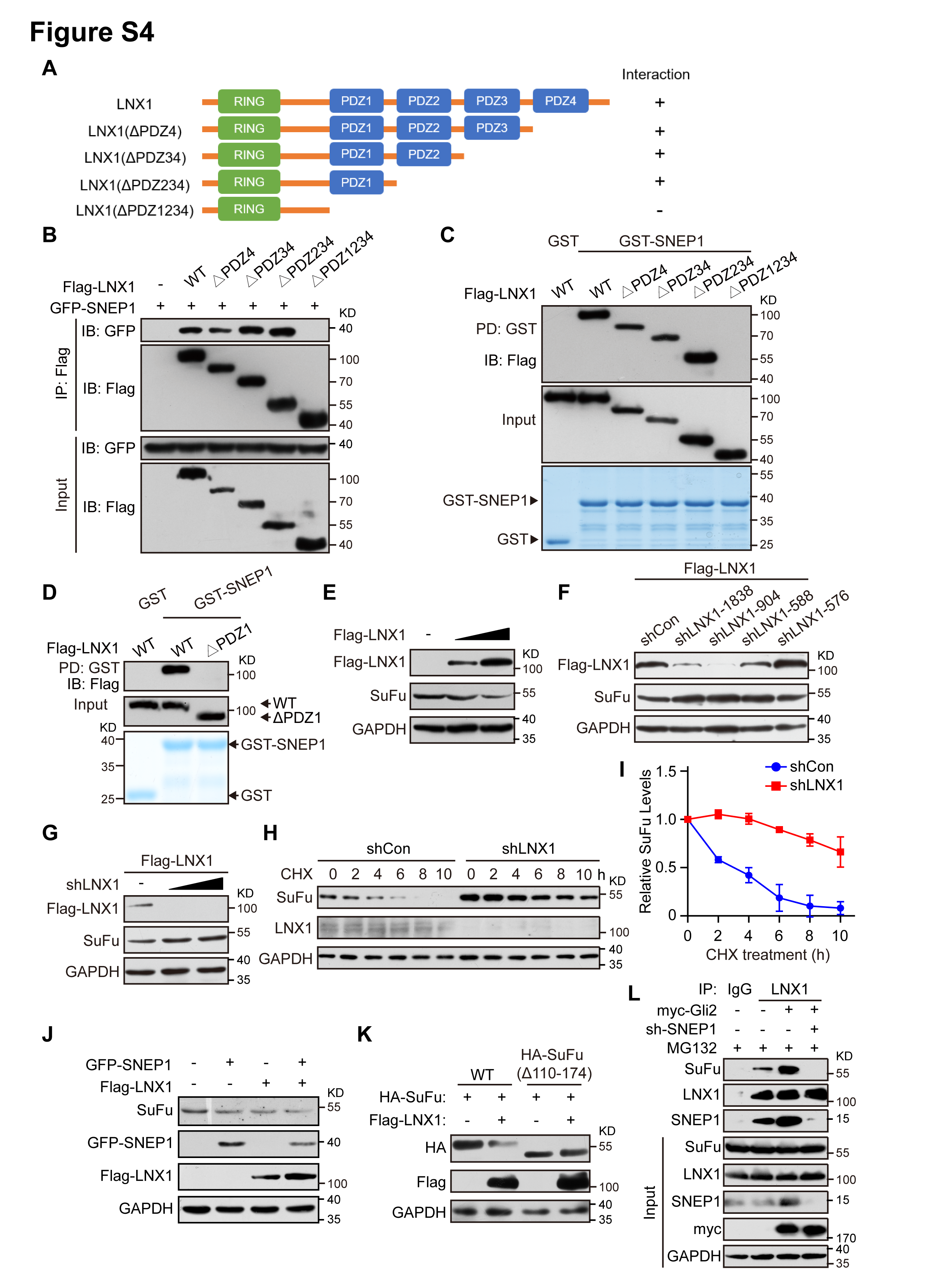

Supplement: Supplementary file 5 — Supplementary Figure 4 [file 41419_2021_3487_MOESM5_ESM.tif]

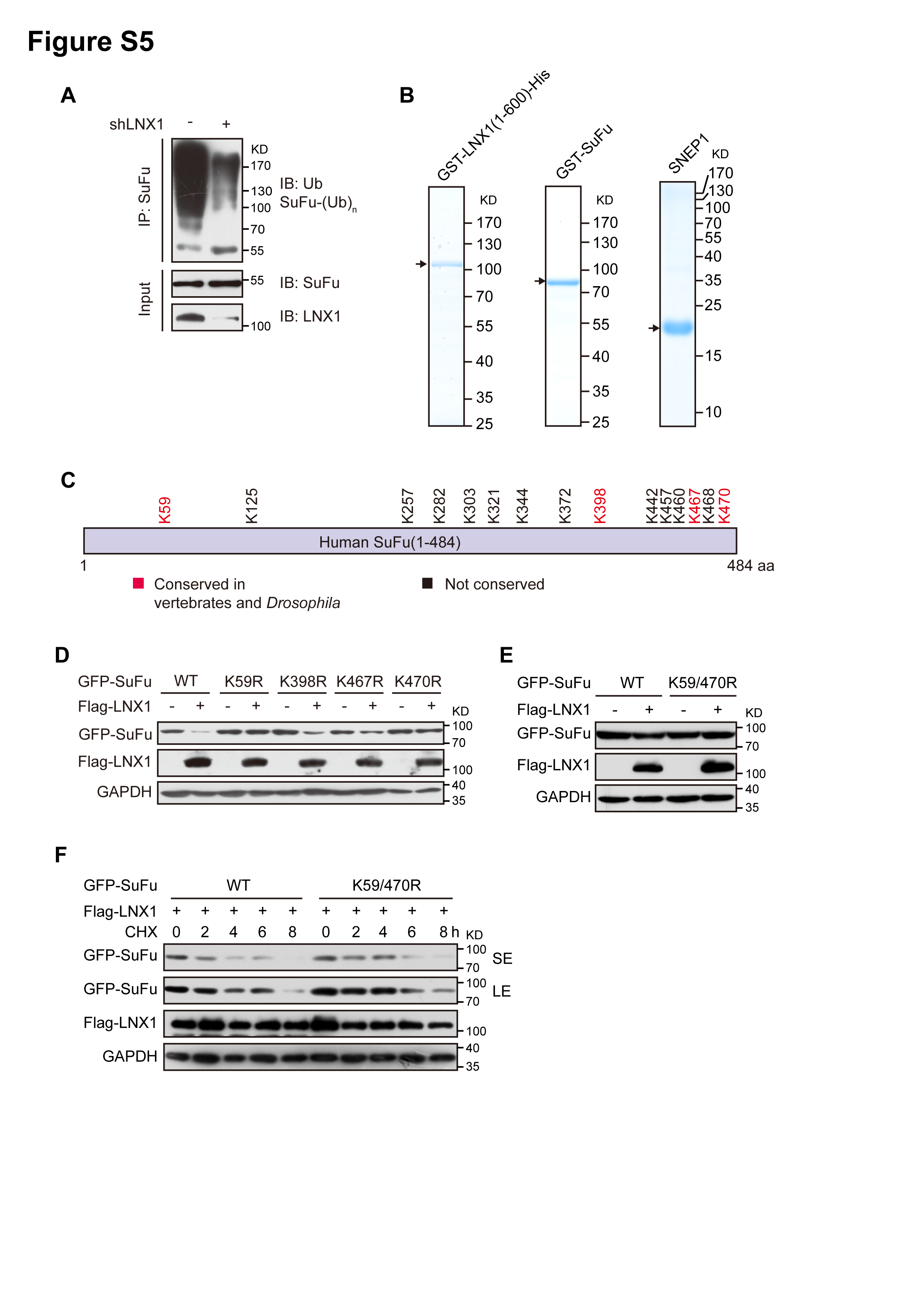

Supplement: Supplementary file 6 — Supplementary Figure 5 [file 41419_2021_3487_MOESM6_ESM.tif]

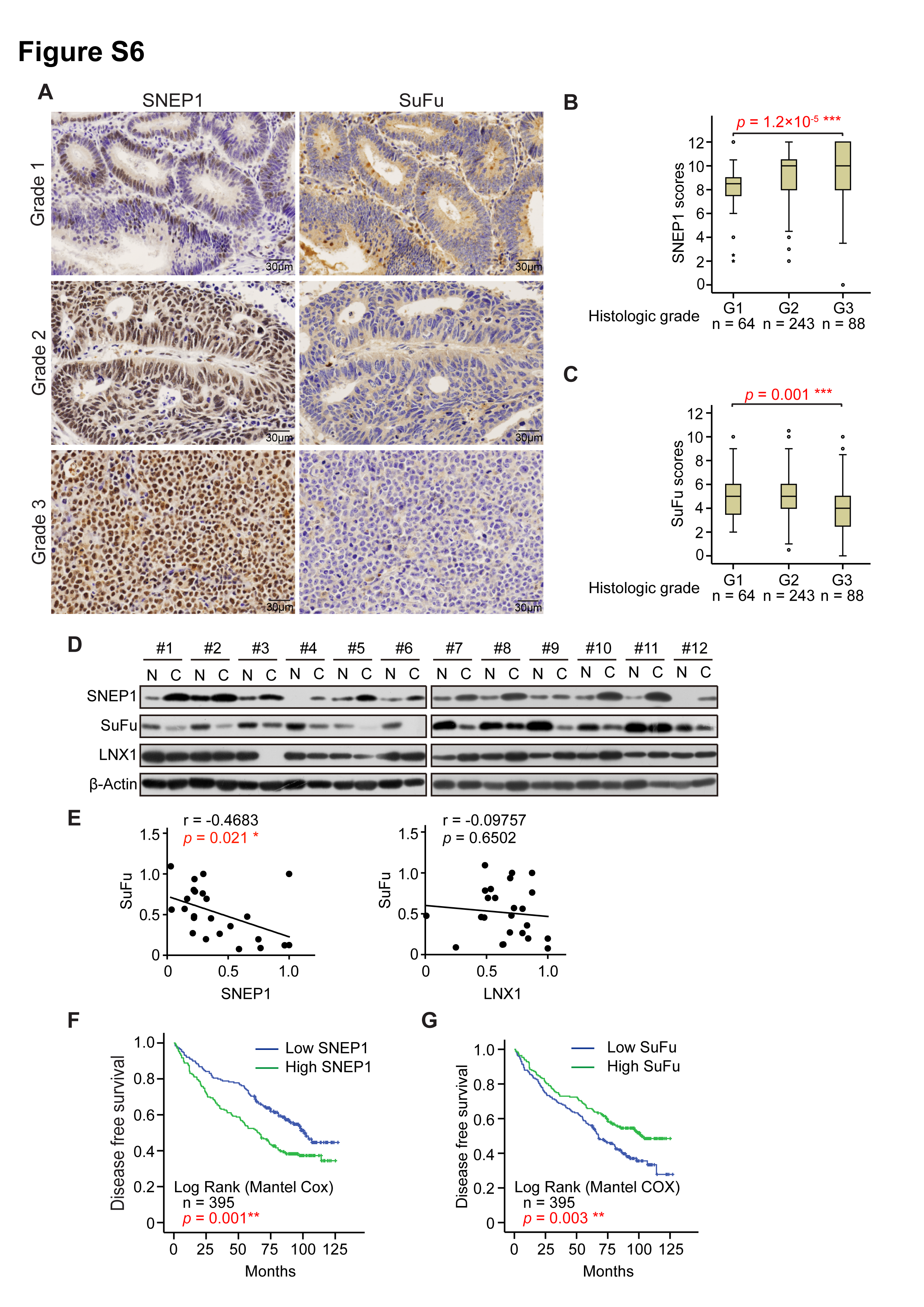

Supplement: Supplementary file 7 — Supplementary Figure 6 [file 41419_2021_3487_MOESM7_ESM.tif]
