# Supplementary material for: Positive feedback of SuFu negating protein 1 on Hedgehog signaling promotes colorectal tumor growth
Source: Cell Death Dis. 2021 Feb 19;12(2):199. doi: 10.1038/s41419-021-03487-0 (PMC7896051; doi:10.1038/s41419-021-03487-0)
Supplement: Supplementary file 8 — Supplementary Table [file 41419_2021_3487_MOESM8_ESM.docx]

**Appendix Table S1: shRNAs or miRNAis used for knockdown of specific genes**

|  | Target sequences (5’-3’) | Start |
| --- | --- | --- |
| shRNA-SNEP1(#1) | 5’-CGCAGGATTGAGGTTAGGACT-3’ | 277 |
| shRNA- SNEP1(#2) | 5’-GCAGGATTGAGGTTAGGACTA-3’ | 278 |
| shRNA- SNEP1(#3) | 5’-AGGATTGAGGTTAGGACTAAA-3’ | 280 |
| shRNA-LNX1(#1838) | 5’-GTGCTTGTATAACTGTAAA-3’ | 2178 |
| shRNA-LNX1(#904) | 5’-CAGGAGACATCATTCTAAA-3’ | 1244 |
| shRNA-LNX1(#588) | 5’-ACTATTAGAAGCAGATCAT-3’ | 928 |
| shRNA-LNX1(#576) | 5’-TTTGAGAGATCCACTATTA-3’ | 916 |

**Appendix Table S2: Primers used for real-time PCR analyses**

| Genes | Forward primer (5’ to 3’) | Reverse primer (5’ to 3’) |
| --- | --- | --- |
| SNEP1 | 5’-GCGCCAGTCACGTCCCTAATG-3’ | 5’-AGTCCTAACCTCAATCCTGCG-3’ |
| SuFu | 5’- CCTTCTGCTAACATCCCCGAG -3’ | 5’- ACGAAAGGTCAACTCAAAGCC -3’ |
| Gli2 | 5’-CAGAATCGCACCCACTCCAACG-3’ | 5’-CGTGGACCGTTTTCACATGCTTCC-3’ |
| Bcl2 | 5’-GCCCTGTGGATGACTGAGTACCTGAAC-3’ | 5’-CAGAGACAGCCAGGAGAAATCAAACAGAG-3’ |
| LNX1 | 5’-GAAGGACAAAGAGCGGGAG-3’ | 5’-ACTCGATTGATCTTGATGCTGG-3’ |
| GAPDH | 5’-CACCAGGGCTGCTTTTAACTCTG-3’ | 5’-GATTTTGGAGGGATCTCGCTCCTG-3’ |
| PTCH1 | 5’-TCGCTCTTGGTGTTGGTGTGGATG-3’ | 5’-CTGTGACATTGCTGATGGACGTGAGG-3’ |

**Appendix Table S3: Primers used for ChIP analyses**

| Gene | Predictive BS (5’ to 3’) | Forward primer (5’ to 3’) | Reverse primer (5’ to 3’) |
| --- | --- | --- | --- |
| SNEP1 | BS1: 5’- TTGGTGTTG-3’ | 5’-CACAGTGAGATGGTGGCGAGG-3’ | 5’-ACAAGCGAGTGCGGATGAACG-3’ |
|  | BS2: 5’- TGGAGGTG-3’ | 5’-CCCACGCCAATCAGCCACAGC-3’ | 5’-CCTAAGGCCACACCGTCCTGC-3’ |
|  | BS3: 5’- GGGGTGGGC-3’ | 5’-GGCTCACACACCTTCGAGT-3’ CC | 5’-ATCCCCACGAACCAGCTTTCC-3’ |

BS: Binding Sequences.

**Appendix Table S4: Primers used for construction of luciferase reporter plasmids**

| Promoters | Range | Forward primer (5’ to 3’) | Reverse primer (5’ to 3’) |
| --- | --- | --- | --- |
| pSNEP1-full（M1） | -1065~-140 | 5’-CCACACAGTGAGATGGTGGCGAGG-3’ | 5’-ATCCCCACGAACCAGCTTTCC-3’ |
| pSNEP1-frag1  （M2） | -1065~-344 | 5’-CCACACAGTGAGATGGTGGCGAGG-3’ | 5’-CCTAAGGCCACACCGTCCTGC-3’ |
| pSNEP1-frag2  （M3） | -1065~-842 | 5’-CCACACAGTGAGATGGTGGCGAGG-3’ | 5’-ACAAGCGAGTGCGGATGAACG-3’ |
| pSNEP1-frag3  （M4） | -602~-140 | 5’-CCCACGCCAATCAGCCACAGC-3’ | 5’-ATCCCCACGAACCAGCTTTCC-3’ |
| pSNEP1-frag4  （M5） | -419~-140 | 5’-GGCTCACACACCTTCGAGTCC-3’ | 5’-ATCCCCACGAACCAGCTTTCC-3’ |
